# Supplementary material for: Write cycling endurance exceeding 1010 in sub-50 nm ferroelectric AlScN
Source: Nat Commun. 2026 Jan 9;17:1507. doi: 10.1038/s41467-025-68221-2 (PMC12890956; doi:10.1038/s41467-025-68221-2)
Supplement: Supplementary file 1 — Supplementary Information [file 41467_2025_68221_MOESM1_ESM.pdf]

Supplementary Information for

Write Cycling Endurance Exceeding 10^{10} in Sub-50 nm Ferroelectric AlScN

Hyunmin Cho[1]†, Yubo Wang[1], Chloe Leblanc[1], Yinuo Zhang[1], Yunfei He[1], Zirun Han[1], Xiaolei Tong[1], Vidhu D. Bulumulla[1], Jonathan Tan[1], Roy H. Olsson III[1]*, Deep Jariwala[1]*

[1]Department of Electrical and Systems Engineering, University of Pennsylvania, Philadelphia, Pennsylvania 19104, USA

*Corresponding author: Deep Jariwala (email: dmj@seas.upenn.edu), Roy H. Olsson III (email: rolsson@seas.upenn.edu)

Supplementary Figure S1-S19

Supplementary Table S1

Supplementary Note 1-5

Supplementary References

Supplementary Figures

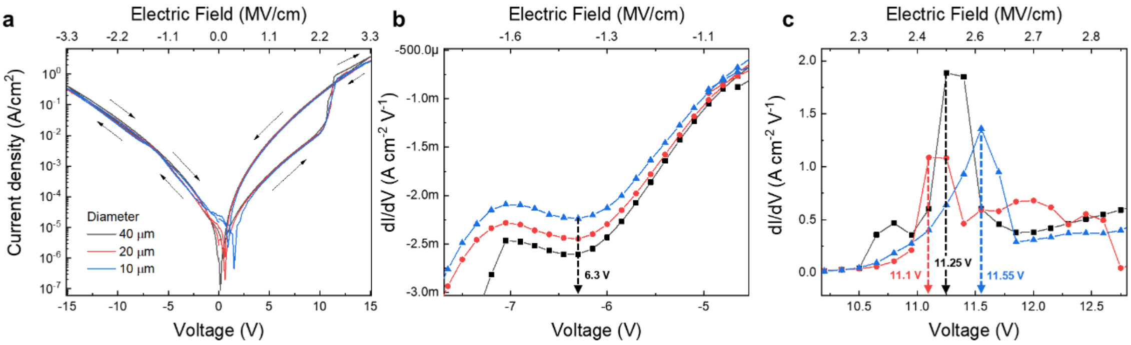

Supplementary Figure S1 | Quasi-direct current density versus voltage (DC-IV) characteristics. (a) The y-axis represents the current density, calculated as the current divided by the sample area for different diameters. **(b)** and **(c)** show the first derivative of the current density with respect to voltage, which is used to extract the coercive voltage (V_C).

As shown in **Supplementary Figure S1a**, no significant variation was observed among different diameters, indicating high reliability with minimal dependence on capacitor area, consistent with the results in **Figure 1d** and **e**. To determine the V_C from the plots in **Supplementary Figure S1a**, we calculated the first derivative of the current density with respect to voltage. The V_C was identified as the voltage at which this derivative reaches a local maximum, corresponding to the point where the rate of change in current density is maximized. **Supplementary Figure S1b** and **S1c** represent the current responses under negative and positive voltage applications, respectively, from which the V_C were extracted for each case. Moreover, the negative coercive voltage remains unchanged regardless of diameter. Although the positive coercive voltage exhibits some variation, these differences arise from measurement errors induced by high leakage.

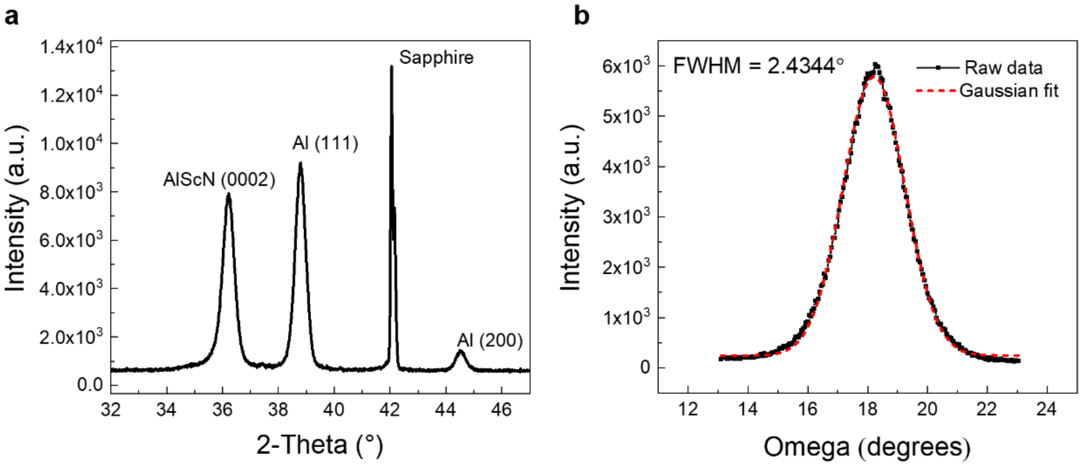

Supplementary Figure S2 | XRD of $Al_{0.64}Sc_{0.36}N$ on Al/Sapphire substrate. (a) θ/2θ scan showing 50 nm Al (111) and 45 nm $Al_{0.64}Sc_{0.36}N$ (0002) diffraction peaks. **(b)** Rocking curve (ω-scan) of $Al_{0.64}Sc_{0.36}N$ (0002).

X-Ray diffraction (XRD) was performed on a Rigaku SmartLab SE. Copper K alpha (Cu Kα) radiation with a wavelength of 1.540 Å. The two-theta (2θ) range was 20˚ to 80˚ with a step size of 0.01˚ and a scan speed of 3˚ per minute.

Supplementary Figure S2a shows the θ/2θ XRD scan data. The scan reveals the θ/2θ peaks corresponding to $Al_{0.64}Sc_{0.36}N$ (0002), Al (111), and Sapphire. The AlScN peak at 36.22° is consistent with previous reports, confirming the high quality of our 45 nm AlScN ferroelectric film[1-6]. The exclusive presence of the (0002) peak indicates that most grains have the c-axis oriented AlScN[1-6]. From the Al (111) peak, we verify that AlScN could be grown on a favorable template established by the Al bottom electrode[1, 3, 7]. The absence of additional AlScN reflections further supports the single orientation of our AlScN[4, 8]. In addition, using a single effective wavelength of 1.540 Å the (0002) peak gives d_{0002} of 2.477 Å and c of 4.954 Å, which is consistent with other reports[1, 4].

Supplementary Figure S2b shows the rocking curve of the AlScN sample. In **Supplementary Figure S2b**, the black scatter points represent the experimental data, and the red dashed line indicates the Gaussian fit. The full width at half maximum (FWHM) of the rocking curve is 2.43° in 45 nm thin AlScN, comparable to values in previous reports[1]. This result also confirms that our 45 nm AlScN film has sufficient crystalline quality for device fabrication and testing.

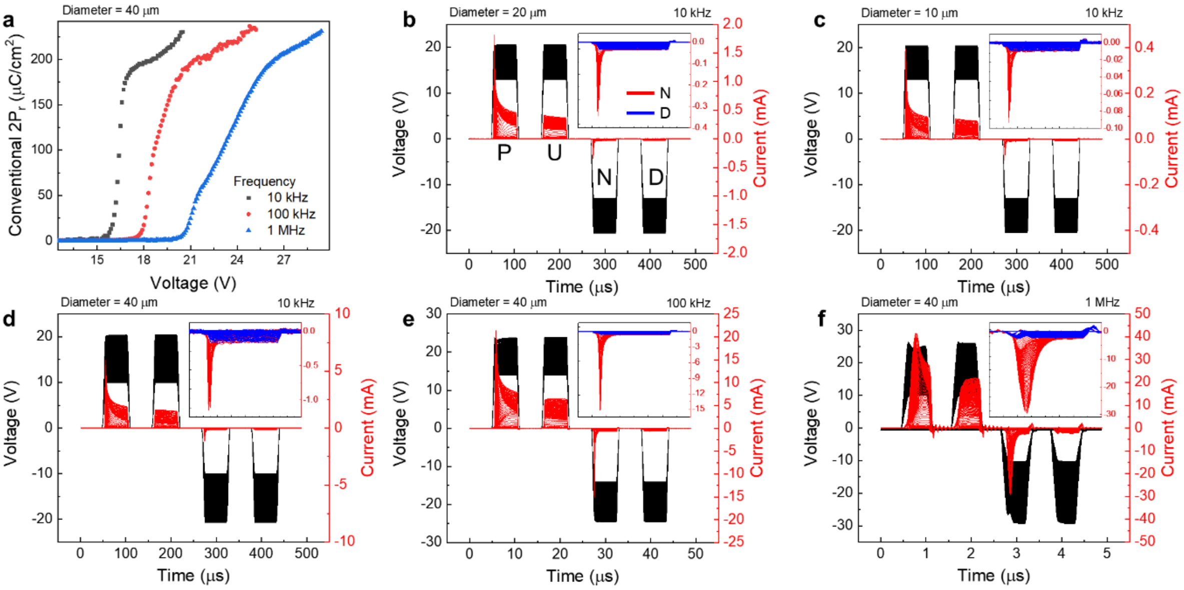

Supplementary Figure S3 | PUND measurements under various conditions. (a) Evolution of conventional $2P_r$ values as a function of applied voltage across varying PUND frequencies, with voltage increments of 0.1 V step. **(b–f)** show detailed current and voltage characteristics across different capacitor diameters and frequency settings. The insets in each plot highlight the difference between N and D pulses by overlaying their respective current responses. The red and blue curves represent the current responses measured when applying the N and D pulses, respectively. **(b–d)** present detailed PUND results explaining each graph in **Figure 1e**, providing a clearer interpretation of the observed responses. Similarly, **(d–f)** present detailed PUND results corresponding to **Supplementary Figure S3a**, providing a clearer view of frequency-dependent behavior in 40 μm diameter capacitors.

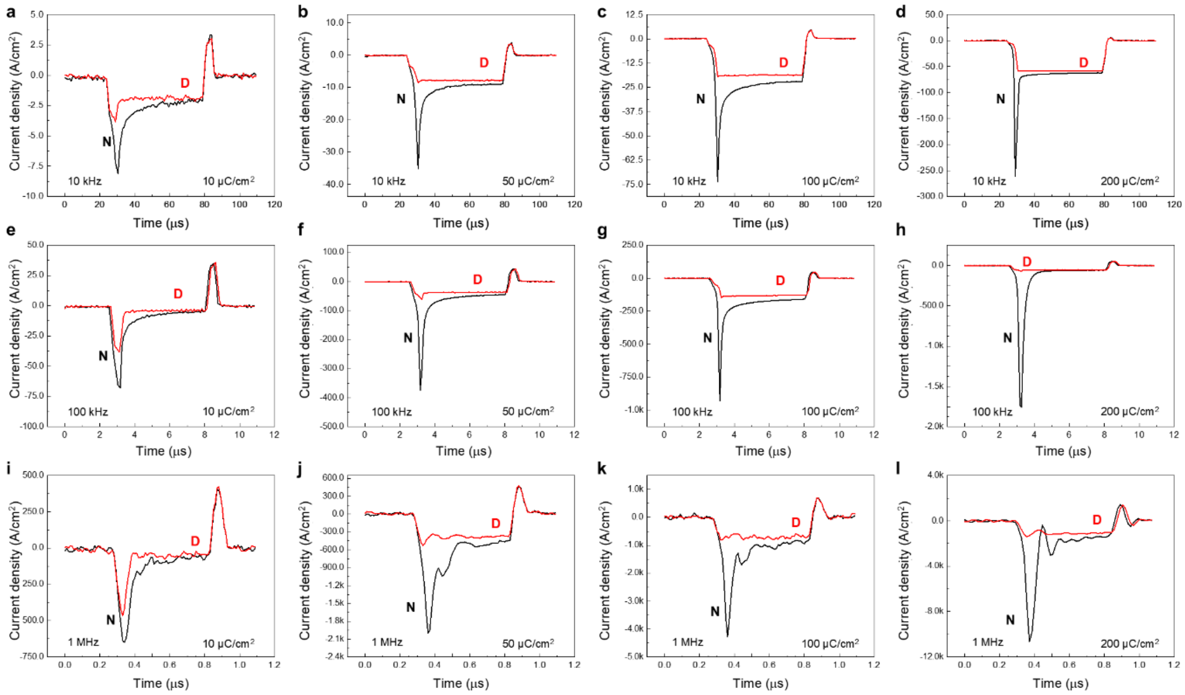

Supplementary Figure S4 | Overlaid current density responses correspond to the difference between N and D voltage pulses during the endurance test. These results are based on PUND measurements taken before leakage started during the endurance test. The data is arranged with increasing frequency in the lower rows and increasing conventional $2P_r$ in the rightward columns. A clear trend shows that higher frequencies lead to an increase in the peak current density, requiring a higher operating voltage to maintain the consistent conventional $2P_r$. Additionally, within the same frequency (same row), moving toward higher conventional $2P_r$ (rightward columns) results in larger current density peaks. This is because, achieving a higher conventional $2P_r$ at a given frequency requires a larger applied voltage, leading to a higher current response. In all cases, the current responses consistently validate the ferroelectric characteristics, confirming the presence of partial polarization in AlScN.

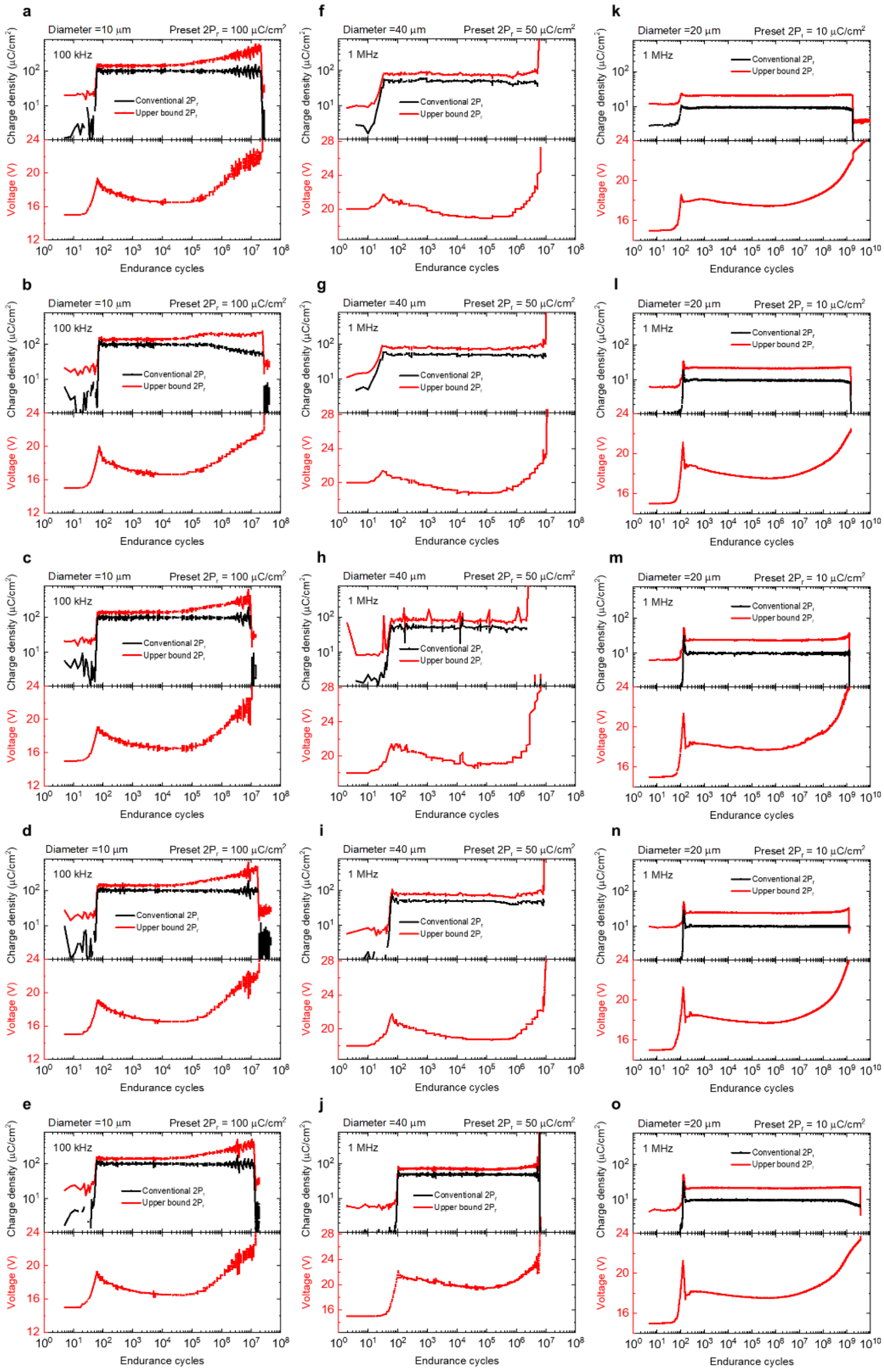

Supplementary Figure S5 | Additional endurance test data under various conditions. (a–e) show the results for 10 μm diameter capacitors measured at 100 kHz, where the conventional $2P_r$ is maintained at 100 μC/cm^2. **(f–j)** present the results for 40 μm diameter capacitors measured at 1 MHz, with the conventional $2P_r$ maintained at 50 μC/cm^2. **(k–o)** display the results for 20 μm diameter capacitors measured at 1 MHz, with the conventional $2P_r$ maintained at 10 μC/cm^2.

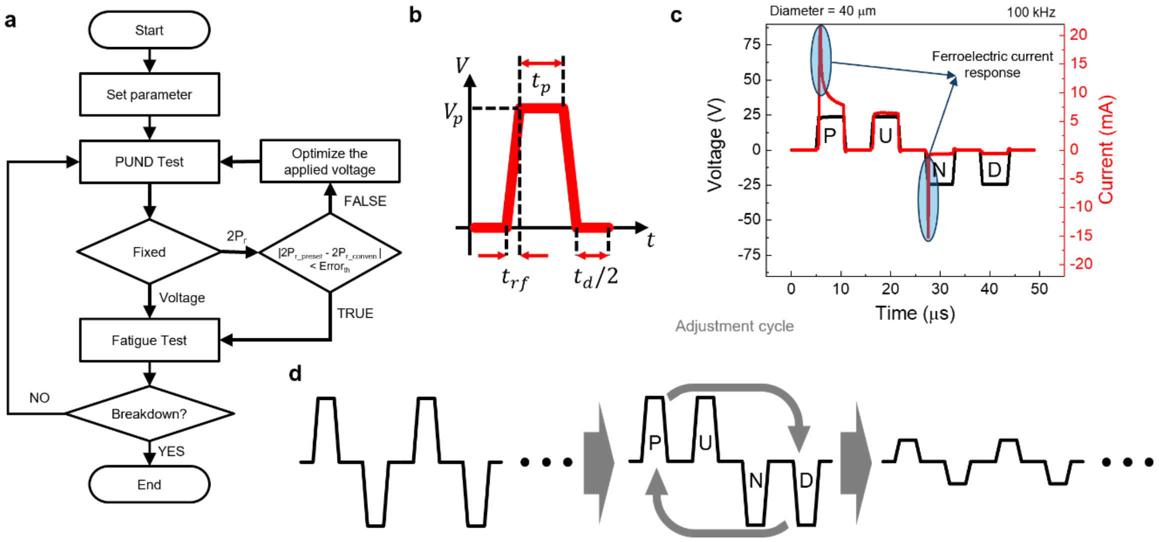

Supplementary Figure S6 | Algorithm for self-adjusting voltage. (a) Overall flowchart for the test. **(b)** Single-pulse configuration with relevant time and voltage parameters, including voltage pulse amplitude (V_p), pulse width (t_p), rise/fall time (t_{rf}), and delay time (t_d). The reason a single pulse only has $t_d/2$ instead of t_d is that the following pulse contributes $t_d/2$, ensuring the full delay time. The frequency is defined as $1/(2 \times t_p)$. **(c)** Detailed PUND measurement results. The blue ellipses in the plots indicate the ferroelectric current response. Other current responses are attributed to leakage and RC delay currents. To isolate the ferroelectric response in PUND measurements, the charge calculated from P and N is subtracted by the charge from U and D (P−U and N−D). The extracted values are then normalized by the capacitor area to obtain the charge density. **(d)** illustrates the pulse train configuration for the fatigue and PUND tests. The first pulse train alternates between positive and negative identical pulses applied to the capacitor. After a certain number of cycles, a PUND measurement is conducted to maintain a stable conventional $2P_r$ by adjusting the applied voltage amplitude. Once the PUND adjustment is completed, the next fatigue pulse train proceeds with the adjusted voltage amplitude for a specified number of cycles.

The algorithm which is performed during the adjustment cycle dynamically adjusts the learning rate for stable voltage regulation. Using real-time feedback, it fine-tunes voltage to align with a target value ($2P_{r\_preset}$), minimizing oscillations and ensuring rapid stabilization. The process begins by analyzing the 2nd derivative of voltage with respect to error ($|2P_{r\_preset} - 2P_{r\_conven.}|$). A negative curvature is preferred to maintain stability in AlScN phase transitions. However, early-phase fluctuations may cause abrupt error reversals, requiring aggressive learning rate adjustments to prevent unnecessary oscillations. Once refined, the algorithm modifies the voltage using a scaled hyperbolic tangent (tanh) function, ensuring smooth, controlled adjustments. The tanh function prevents extreme corrections while maintaining

sensitivity to error trends. This cycle repeats until stability is achieved. A final assessment ensures consistency, eliminating fluctuations.

PUND measurements were conducted to quantify the amount of polarization switching before and after fatigue testing. However, the reliability of this method becomes questionable when operating in the partial switching regime. During the P and U steps, polarization switching occurs twice before the application of the N and D pulses, which differs from the actual fatigue condition involving a single switching event per cycle. The switched polarization during the N and D pulses strongly depends on the initial state defined by the preceding P and U pulses. Since this state is different from the one in fatigue testing, particularly under partial switching, the extracted values from PUND measurements are not directly representative of the true fatigue-induced switching behavior. Thus, PUND cannot accurately resolve the switching characteristics in this regime. Despite these limitations, PUND was employed in this study to maintain consistency with prior reports and enable a comparative analysis within this work. A more accurate evaluation of partial polarization switching will require further methodological refinement and validation.

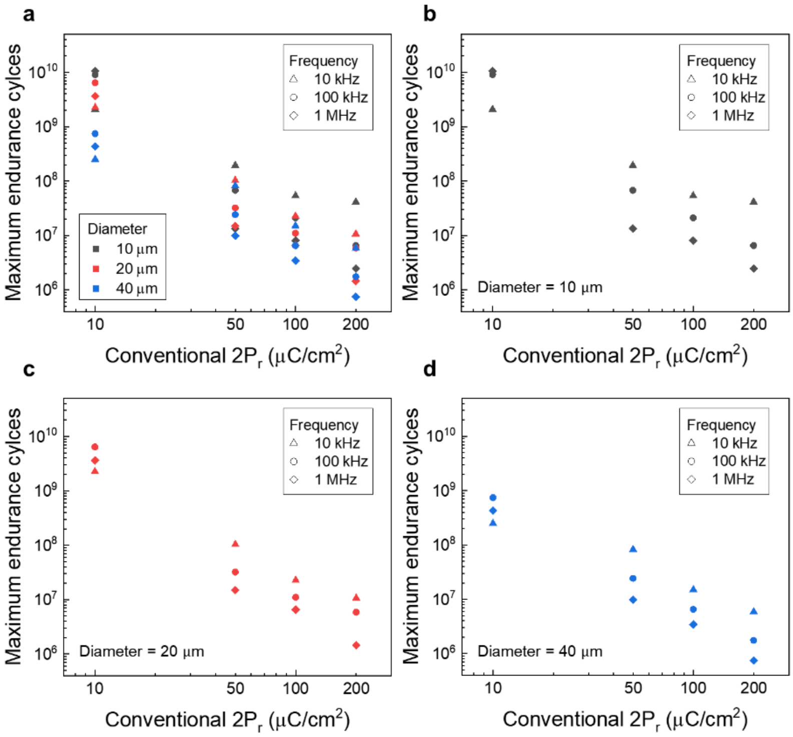

Supplementary Figure S7 | Reorganized plots representing relationships between conventional 2P$_r$ and endurance. (a) Comprehensive dataset covering all conditions. Subdivided plots for different diameters: **(b)** 10 μm, **(c)** 20 μm, and **(d)** 40 μm.

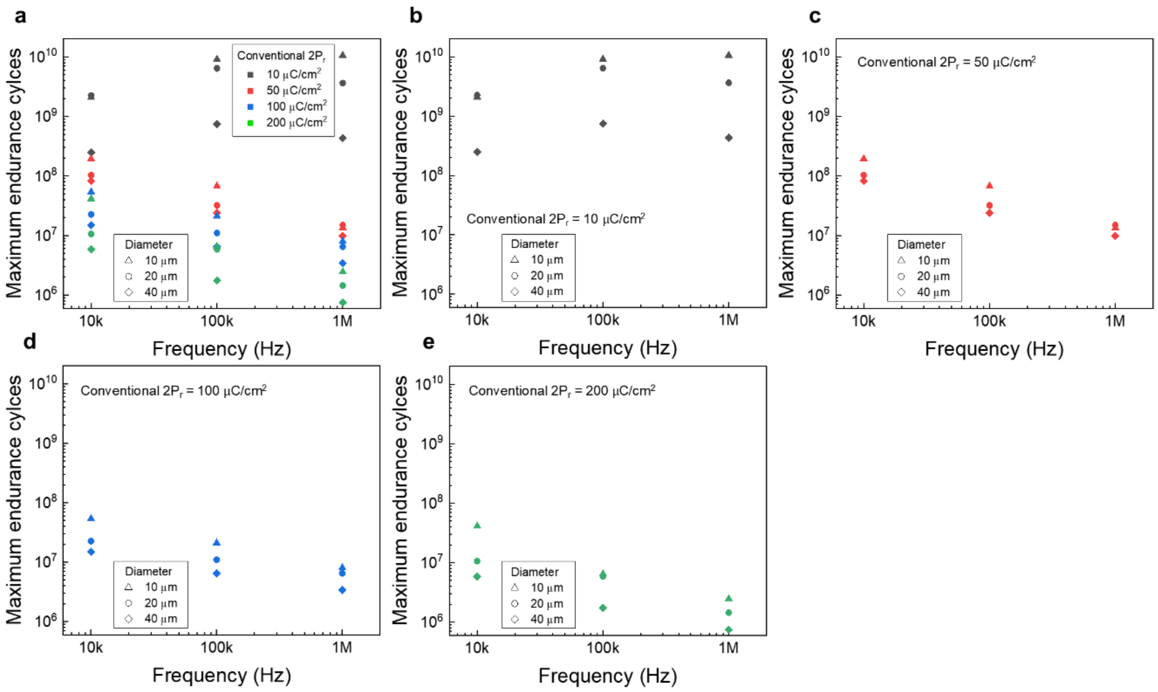

Supplementary Figure S8 | Reorganized plots representing relationships between frequency and endurance. **(a)** Comprehensive dataset covering all conditions. Subdivided plots for maintained conventional $2P_r$: **(b)** 10 μC/cm^2, **(c)** 50 μC/cm^2, **(d)** 100 μC/cm^2, and **(e)** 200 μC/cm^2.

The distinct endurance trends as a function of preset $2P_r$ originate from the competition between two mechanisms. First, the coercive voltage increases with increasing frequency, imposing higher operating stress that accelerates degradation. Second, the pulse width shortens with frequency, which limits the exposure time to high electric fields and mitigates breakdown. At high preset $2P_r$ values, the stress effect dominates, leading to reduced endurance. In contrast, under low partial switching conditions (≈10 μC/cm^2), the reduced stress exposure governs and results in the opposite frequency dependence. Further theoretical and experimental investigations are needed to clarify the interplay between electrical stress and endurance in wurtzite ferroelectrics.

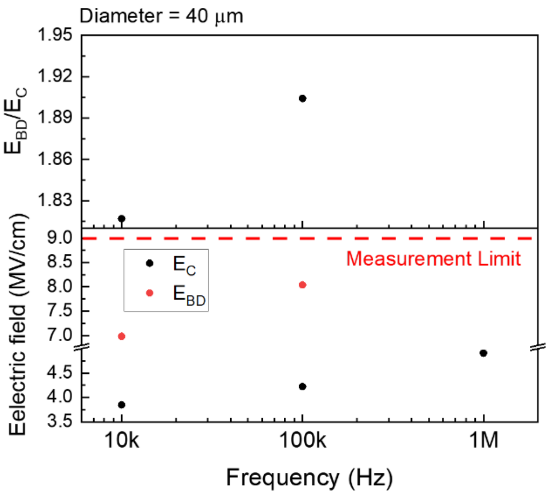

Supplementary Figure S9| Frequency Dependence of Coercive and Breakdown Electric Fields. This plot illustrates the correlation between the operation frequency in PUND measurements and key ferroelectric properties, specifically the coercive electric field and the breakdown electric field (bottom panel), as well as their ratio (top panel). The red dashed line indicates the operational limit of our measurement system. Consequently, obtaining the breakdown voltage for the 1 MHz case is impossible within the constraints of this setup.

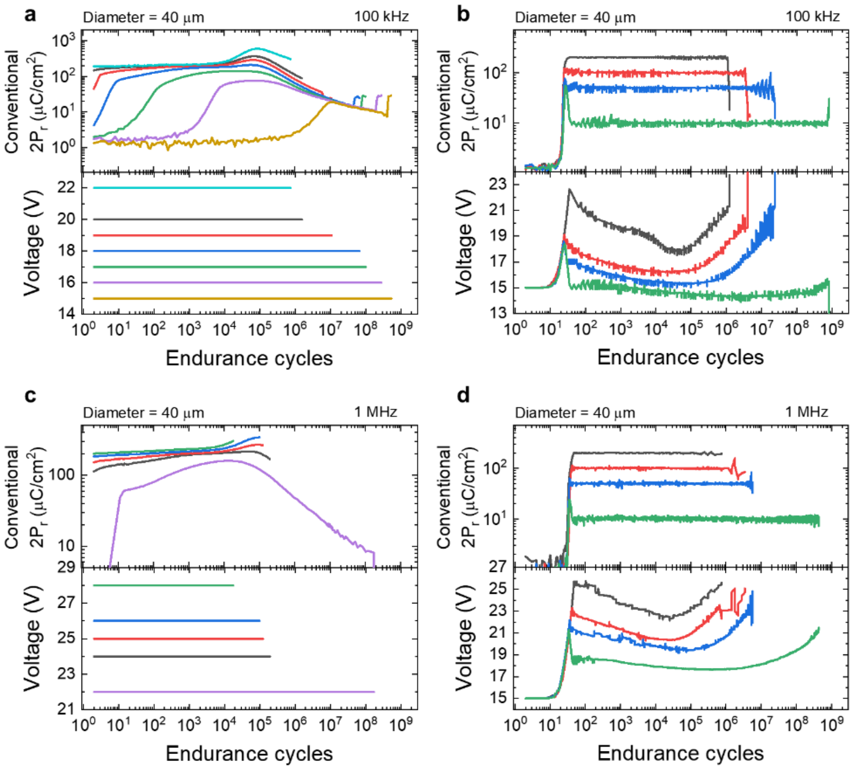

Supplementary Figure S10 | Endurance test results under various frequency conditions. Endurance cycles were conducted with **(a)** and **(c)** a constant applied voltage pulse, while **(b)** and **(d)** used an adjusted applied voltage pulse to maintain the conventional $2P_r$ close to preset $2P_r$.

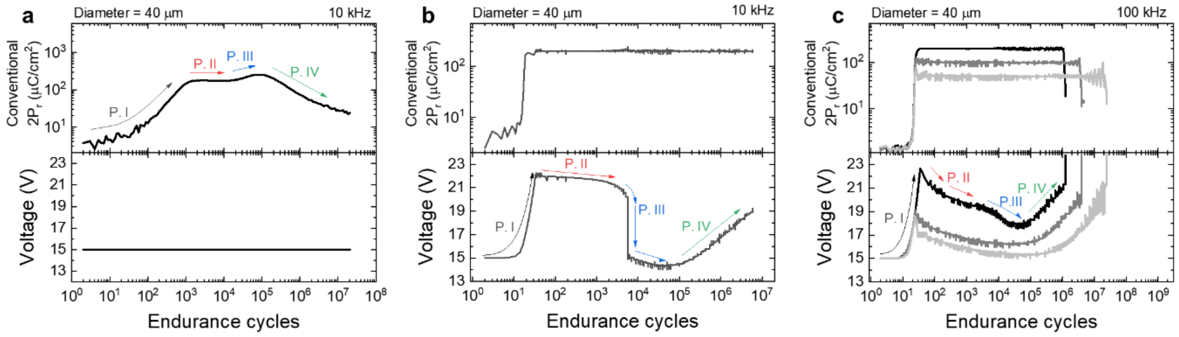

Supplementary Figure S11 | Phase evolution during endurance test. Data was obtained from a 40 μm capacitor. **(a)** shows phase evolution for 15 V at 10 kHz. The arrows mark phase changes through variations in $2P_r$. **(b)** adjusts the voltage to keep the conventional $2P_r$ at 200 μC/cm^2, tracking phase shifts through applied voltage variations. **(c)** shows the results with varying frequency and under conditions that maintain an even lower conventional $2P_r$, ensuring the existence from wake up to reduce degradation effects.

Each phase (P.) in **Supplementary Figures S11 a–c** indicates distinct polarization phases. A comprehensive analysis is provided in **Supplementary Note 5**. In **Supplementary Figure S11a**, P. I corresponds to the wake-up process, P. II represents the stabilized ferroelectric state, P. III is dominated by leakage currents, and P. IV signifies the onset of fatigue-induced degradation. In **Supplementary Figure S11b**, P. I involves voltage modulation to establish the optimal amplitude for the conventional $2P_r$, approaching the preset $2P_r$ value of 200 μC/cm^2, along with the initial quick wake-up phase. P. II represents the stabilized ferroelectric state, P. III is characterized by increased leakage current contributions, and P. IV marks the progression of degradation. In **Supplementary Figure S11c**, P. I show the same behavior as in **Supplementary Figure S11b**. However, in P. II, partial wake-up occurs simultaneously with the stable phase, indicating an extended polarization stabilization period. P. III is characterized by increased leakage current contributions, and P. IV marks the progression of degradation effects. This distinction highlights that for lower preset $2P_r$ conditions, the second phase sustains partial wake-up, leading to the coexistence of the stable and wake-up phases, which is absent in the higher preset $2P_r$ case.

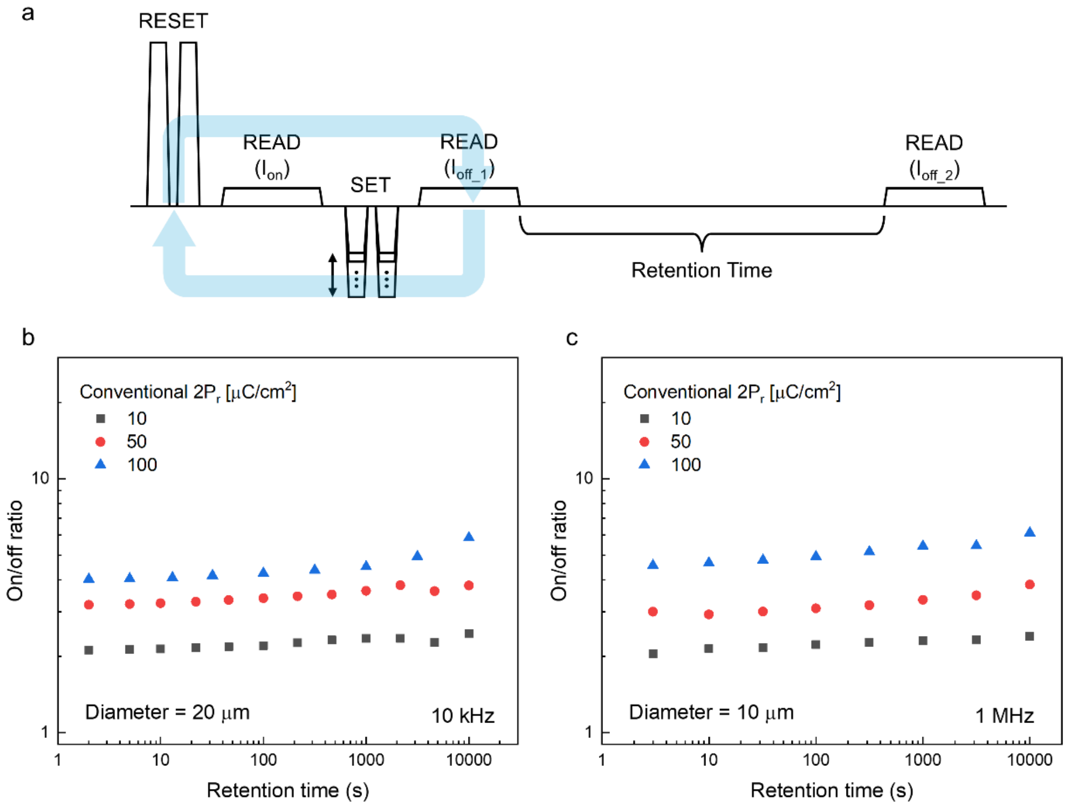

Supplementary Figure S12 | Retention test results under various conditions and device diameters. (a) illustrates the voltage pulse configuration used in the test. **(b)** and **(c)** show the retention performance.

Supplementary Figure S12 presents the retention results under different conditions, including 10 kHz for 20 μm and 1 MHz for 10 μm devices. Retention was measured using a current-based readout at a low voltage of 6 V to verify the state with minimal disturbance. As shown in **Supplementary Figure S12 a**, the test sequence consists of two positive RESET pulses followed by two negative SET pulses, with a read step after each pair to record the current. To initialize the M-polar state, 18 V pulses at 10 kHz were applied, ensuring full switching as confirmed in **Figure 1e**. The conventional $2P_r$ values extracted from the RESET pulses also indicate complete polarization switching. These pulse sequences and read steps were repeated until the conventional $2P_r$ obtained from the SET pulses reached the target $2P_r$. The device was then held for the designated retention time before a final read to assess resistance variation.

The AlScN was first initialized to the M-polar state, followed by partial switching toward the N-polar state to preset $2P_r$ values of 10, 50, and 100 $\mu C/cm^2$. Because conduction in AlScN is polarization

dependent [7], the current after RESET corresponds to the low-resistance state (LRS), while the current after SET corresponds to the high-resistance state (HRS). To eliminate any read-induced effects, RESET and SET were alternated again, and the applied voltage was adjusted until the SET-derived $2P_r$ returned to the target value for a different retention time test. We define I_{on} as the current read after RESET, I_{off_1} as the current after the first SET, and I_{off_2} as the current after the retention interval. Retention is quantified by comparing I_{on}/I_{off_1} and I_{on}/I_{off_2}. If strong depolarization occurred during the waiting time, I_{on}/I_{off_2} would approach one. As shown in **Supplementary Figure S12b** and **c**, this ratio exhibits no significant degradation and remains nearly constant up to 10^4 s, with only a slight increase attributed to partial switching of residual M-polar regions toward the N-polar direction. This minor change is likely due to the preferential imprinting of the intrinsically N-polar state in pristine AlScN. These results confirm robust retention of partially switched states in AlScN, consistent with prior reports on full and multistate switching [9-13].

Partial polarization in AlScN exhibits remarkable stability even under rapid cycling, showing negligible depolarization or relaxation. Its strong domain pinning and low dielectric permittivity suppress back-switching[14,15], ensuring retention remains stable up to 1 MHz. These results demonstrate that AlScN can sustain nonvolatile operation at high speed, highlighting its promise for future embedded and compute-in-memory applications.

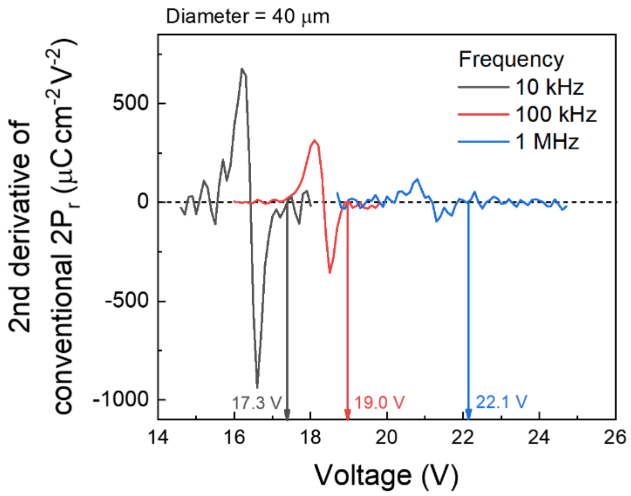

Supplementary Figure S13 | Extraction of the coercive voltage (V_c). V_c is determined through PUND measurements by analyzing the second derivative of the conventional 2P_r. The point where this derivative reaches zero signifies polarization saturation. This method enables more meaningful comparisons, making it well-suited for the PUND-based endurance test used in this work.

The second derivative of conventional 2P_r decreases as frequency increases, aligning with the trend observed in the slope variation of conventional 2P_r against applied voltage, as shown in **Supplementary Figure S3a**.

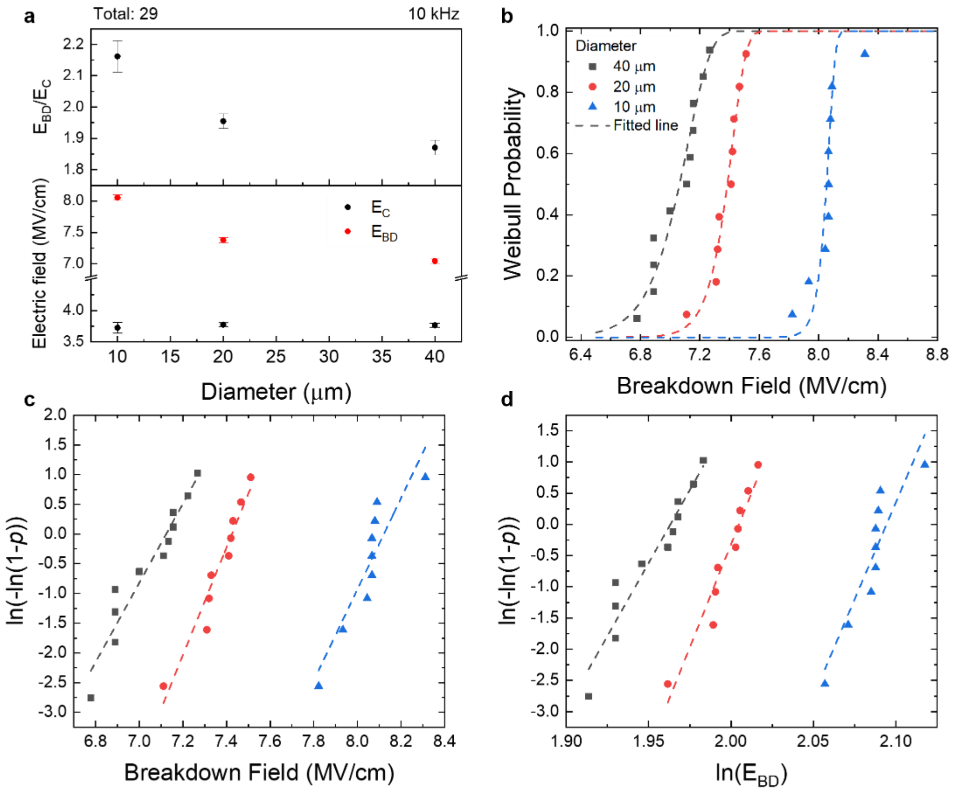

Supplementary Figure S14 | Dependence of electrode diameter on breakdown and coercive fields. (a) Relationship between electrode diameter and ferroelectric parameters including coercive field (E_C, bottom panel), breakdown field (E_{BD}), and their ratio (E_{BD}/E_C, top panel). **(b)** Weibull probability plots of breakdown field for different diameters. **(c, d)** Reorganized Weibull plot and corresponding linearization to visualize the statistical reliability of MFM Breakdown voltage across different sizes. Symbols represent experimental data, and the dashed line indicates the fitted plots.

1

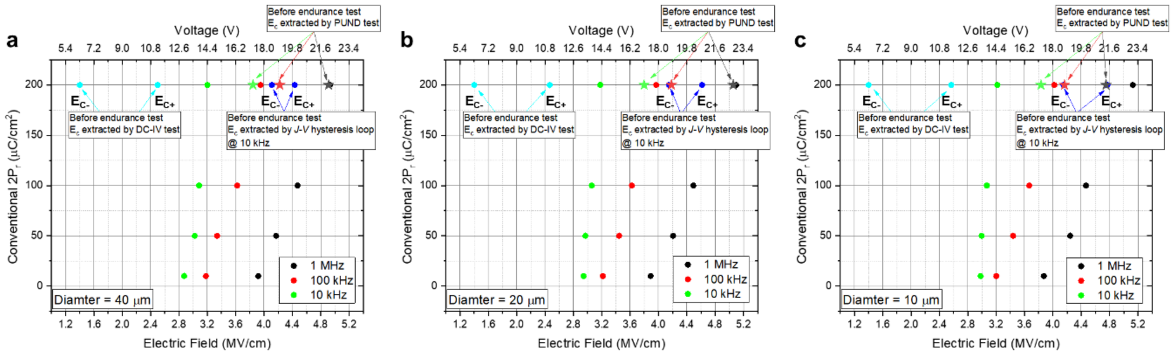

Supplementary Figure S15 | Conventional $2P_r$ as a function of applied voltage and electric field for different device sizes. (a) 40 μm, **(b)** 20 μm, and **(c)** 10 μm diameter devices. Scatter symbols represent different measurement conditions: black, red, and green stars represent conventional $2P_r$ values obtained before the endurance test using the PUND measurement (as shown in **Supplementary Figure S13**) at 1 MHz, 100 kHz, and 10 kHz, respectively. Black, red, and green circles represent the adjusted applied voltage before the leakage phase begins at 1 MHz, 100 kHz, and 10 kHz, respectively. Blue circles represent E_C extracted from *J-V* hysteresis loops (**Figure 1e**) at 10 kHz before the endurance test, and cyan circles represent E_C extracted from DC-IV curves (**Supplementary Figure S1a**). The E_{C-} and E_{C+} indicate results for negative and positive voltage, respectively.

Notably, the change of conventional $2P_r$ against electric field is slower as frequency increases. This suggests that high frequency enables more precise control over polarization switching. In contrast, at low frequency, the steep slope indicates that partial switching is difficult to control.

Although both E_{C-} and E_{C+} were obtained, E_{C-} should be used for analysis to maintain consistency with our endurance test comparisons. As observed here, the E_C extracted from DC-IV (cyan circle) is much lower than that of the *J-V* hysteresis loop (blue circle) or PUND (green circle). This occurs because the E_C generally decreases with lower frequency, and the applied voltage in the DC-IV measurement, being close to 0 Hz, results in the lowest E_C[1, 16-19]. Additionally, the E_{C-} from the *J-V* hysteresis loop (blue circle) and PUND (green star) remains higher than the applied electric field obtained from the endurance test (green circle). This suggests that the wake-up effect lowers the E_C.

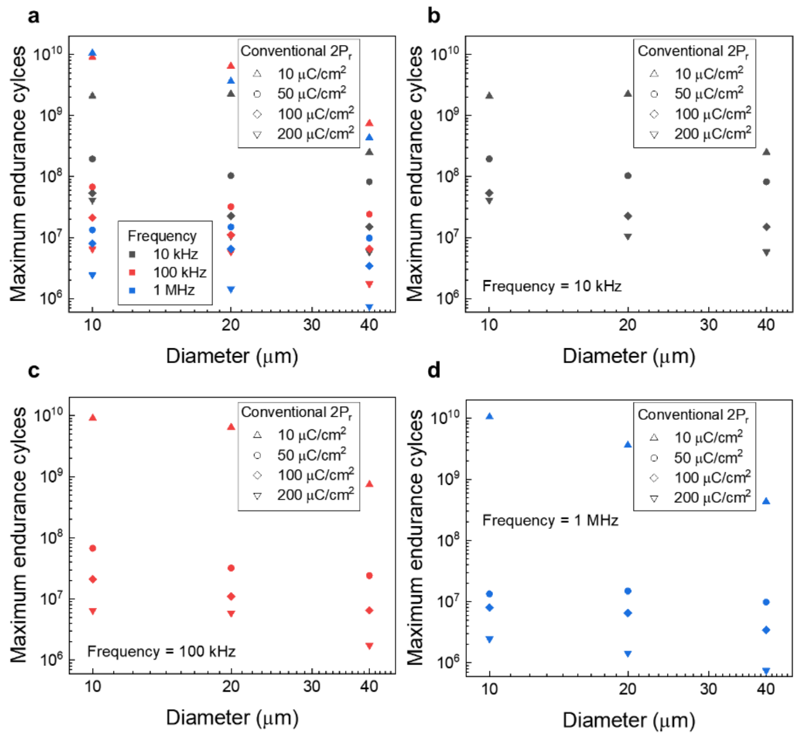

Supplementary Figure S16 | Reorganized plots representing relationships between diameter and endurance. **(a)** Comprehensive dataset covering all conditions. Subdivided plots for different frequency: **(b)** 10 kHz, **(c)** 100 kHz, and **(d)** 1 MHz.

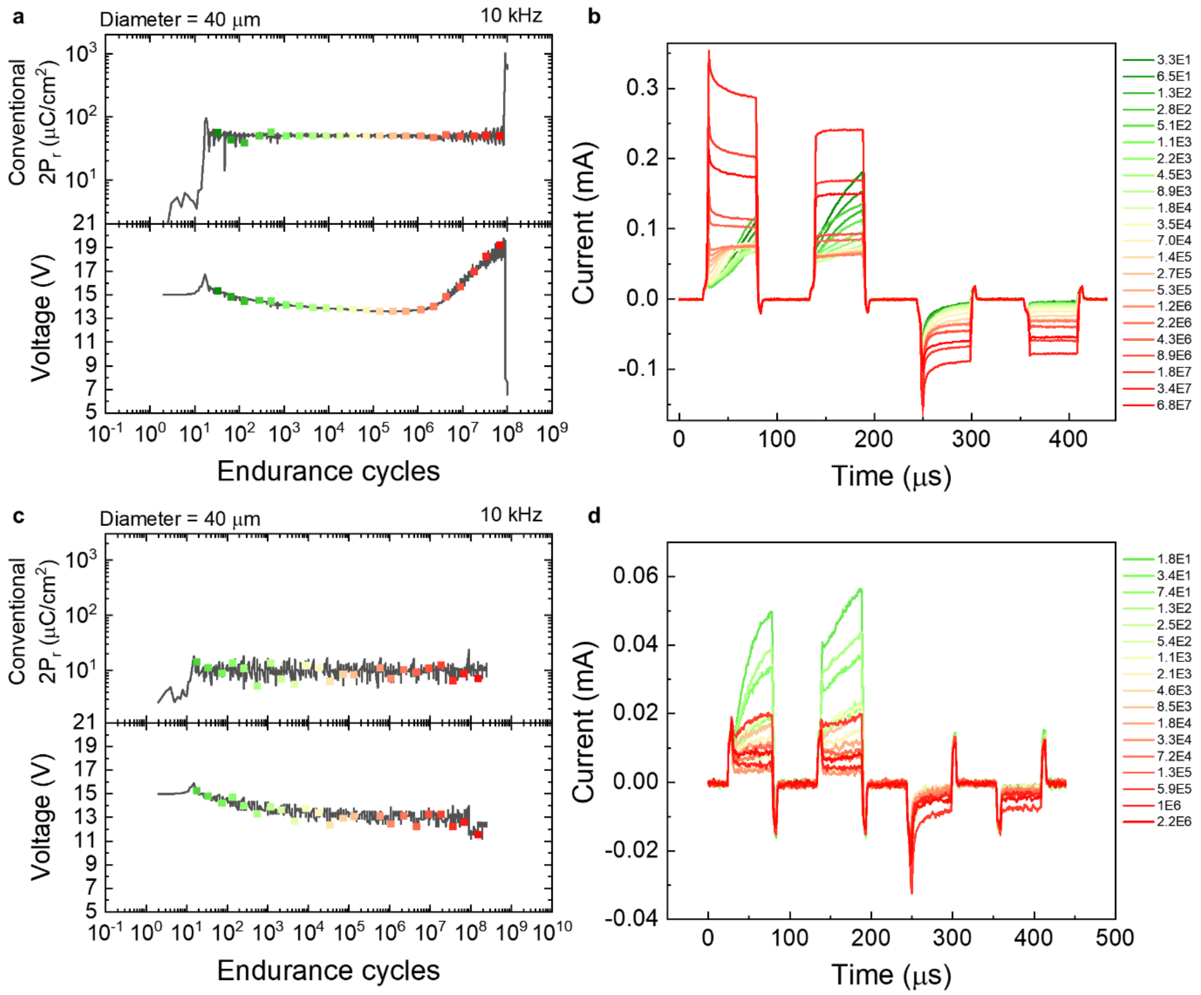

Supplementary Figure S17 | PUND measurement results obtained from endurance cycling. (a) and **(c)** Endurance test result of a capacitor with a 40 μm diameter, maintaining a conventional $2P_r$ value of 50 and 10 μC/cm^2 under a 10 kHz pulse. The rainbow markers correspond to the different plots shown in **(b)** and **(d).** The legend on **(b)** and **(d)** indicates the moment when the PUND measurement was performed.

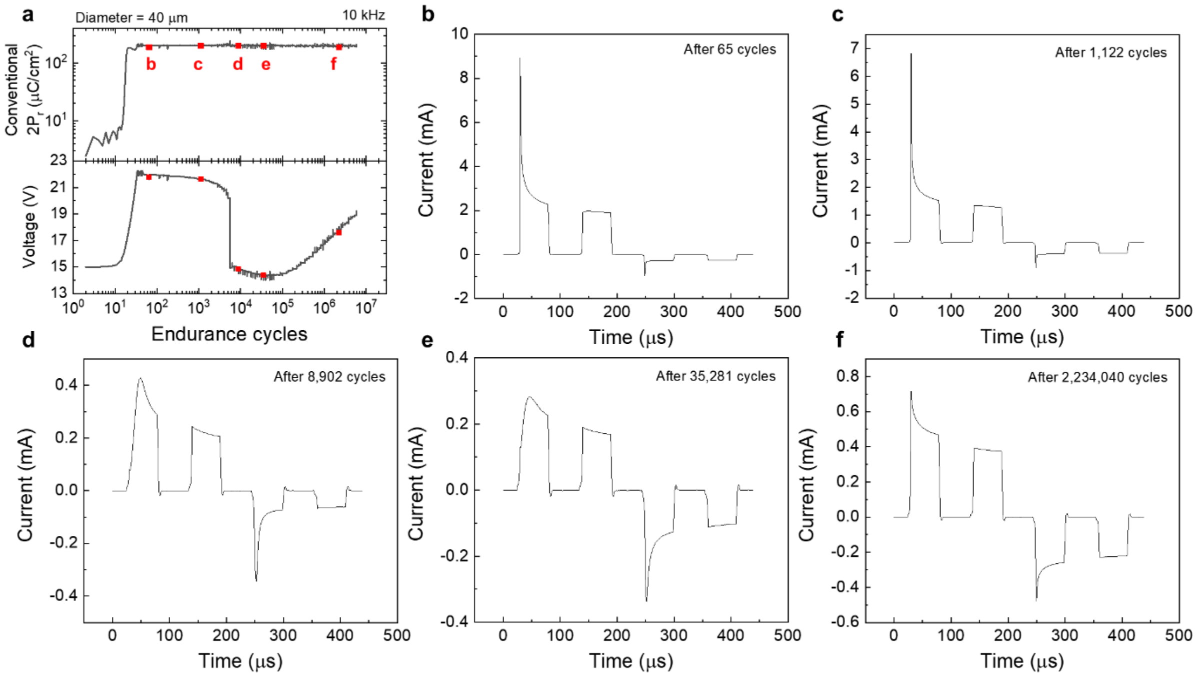

Supplementary Figure S18 | PUND measurement results obtained from endurance cycling. (a) Endurance test result of a 40 μm diameter capacitor, maintaining conventional $2P_r$ at 200 μC/cm^2 under a 10 kHz pulse. The red markers indicate specific cycles corresponding to **(b-f)**, where PUND measurements were conducted to evaluate polarization switching behavior.

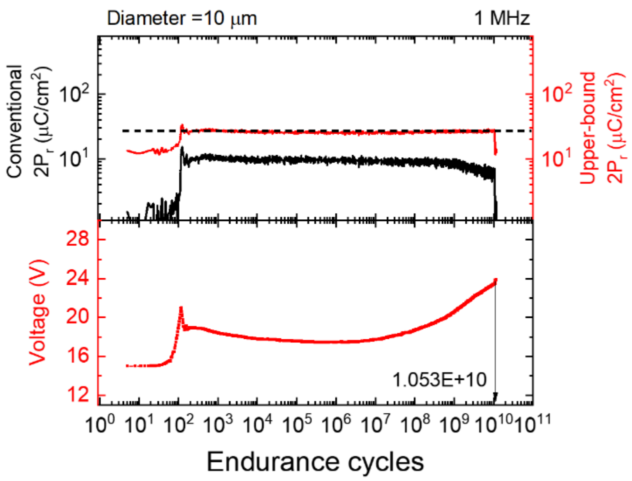

Supplementary Figure S19 | The best endurance test result.

Supplementary Table S1 | Energy Consumption per Cycle for AlScN vs HfO_2 Ferroelectrics.

Material	Thickness & Diameter [nm & μm]	$2P_r$ [μC/cm²]	Frequency	Energy consumption				Ref.
				E_{PN} [nJ]	E_{UD} [nJ]	E_{PN} / ($2P_r$ × Area) [mJ/μC]	E_{UD} / ($2P_r$ × Area) [mJ/μC]	
$Al_{0.67}Sc_{0.36}N$	45 & 10	200	10 kHz	23.19	16.7	0.1477	0.1063	Our works
$Al_{0.67}Sc_{0.36}N$	45 & 10	100	10 kHz	6.67	9.9	0.0849	0.1260	
$Al_{0.67}Sc_{0.36}N$	45 & 10	50	10 kHz	4.02	6.16	0.1022	0.1568	
$Al_{0.67}Sc_{0.36}N$	45 & 10	10	10 kHz	0.16	0.07	0.0200	0.0090	
$Al_{0.67}Sc_{0.36}N$	45 & 10	50	100 kHz	3.84	4.04	0.0978	0.1030	
$Al_{0.67}Sc_{0.36}N$	45 & 10	50	1 MHz	5.12	2.56	0.1302	0.0652	
$Al_{0.67}Sc_{0.36}N$	45 & 40	50	10 kHz	46.32	78.5	0.0738	0.1250	
$Al_{0.72}Sc_{0.28}N$	5 & 10	100	1 MHz	12.57	10.29	0.1600	0.1310	1
$Al_{0.72}Sc_{0.28}N$	5 & 10	250	1 MHz	25.27	19.29	0.1287	0.0983	1
$Al_{0.72}Sc_{0.28}N$	27 & 25	170	1 MHz	406.80	272.78	0.4875	0.3269	1
$Al_{0.72}Sc_{0.28}N$	18 & 25	200	1 MHz	478.15	397.97	0.4870	0.4054	1
$Al_{0.72}Sc_{0.28}N$	10 & 10	200	1 MHz	51.92	47.03	0.3305	0.2994	1
$Al_{0.68}Sc_{0.32}N$	10 & 10	200	1 MHz	68.92	57.39	0.4388	0.3654	20
$Al_{0.7}Sc_{0.3}N$	10 & 1.5	225	1 MHz	0.93	0.73	0.2344	0.1844	21
$Al_{0.7}Sc_{0.3}N$	40 & 110	175	500 kHz	877.72	819.35	0.0528	0.0493	22
$Al_{0.7}Sc_{0.3}N$	40 & 70	140	500 kHz	584.67	207.68	0.1085	0.0386	23
$Hf_{0.5}Zr_{0.5}O_2$	10 & 226	36	1 kHz	98.52	18.71	0.0069	0.0014	24
$Hf_{0.5}Zr_{0.5}O_2$	10 & 113	60	2 kHz	31.8	~ 0	0.0053	~ 0	25
$Hf_{0.5}Zr_{0.5}O_2$	1.5 & 56	13	500 kHz	2.28	2.1	0.0069	0.0062	26
$Hf_{0.5}Zr_{0.5}O_2$	12.3 & 40	16.4	1 MHz	4	0.76	0.0195	0.0037	27
$Zr_{0.33}Hf_{0.67}O_2$	10 & 1.35	10	1 kHz	0.03	0.002	0.1790	0.0140	28
ZrO_2	10 & 113	51	1 kHz	111.22	30.4	0.0218	0.0059	28

To quantify energy consumption, we integrated $(\int V(t) \cdot I(t)dt)$ over each pulse window in PUND measurements, from the onset to the end of the applied voltage. In practice, the energy stored in the device and the energy dissipated during operation should be distinguished. However, due to the lack of detailed material parameters reported in prior studies, such separation could not be made in **Supplementary Table S1**. Nevertheless, we note that this distinction was considered in our followed analysis and interpretation. The four pulse energies are denoted as E_P, E_U, E_N, and E_D. We define $E_{PN} = E_P + E_N$, corresponding to switching windows, and $E_{UD} = E_U + E_D$, corresponding to non-switching

windows. While E_{UD} may still include a small extent of switching contributions under partial polarization switching, it gives an upper-bound estimate of non-switching losses. To prepare the comparison in **Supplementary Table S1**, we collected data from previous reports[20-28]. As in **Supplementary Figure S6** where frequency was calculated from pulse configuration parameters, we also extracted pulse information from prior reports and recalculated the corresponding frequencies using our method to ensure consistency in **Supplementary Table S1**.

In **Supplementary Table S1**, systematic trends were confirmed across all cases. The results show that power consumption increases steadily with $2P_r$. For example, at 45 nm thickness and 10 μm diameter, E_{PN} rises from 0.16 to 23.19 nJ as $2P_r$ increases from 10 to 200 μC/cm^2. E_{UD} also increases with $2P_r$ due to higher applied voltages and leakage contributions.

AlScN tends to show higher energy consumption than HfO_2 based ferroelectric material under similar test conditions. AlScN inherently has a larger $2P_r$, which means more energy is stored in the device. Since the stored energy is also counted as part of the consumed energy in this calculation, the evaluated energy consumption inevitably appears larger. However, in the case of small $2P_r$, the energy consumption of AlScN can approach that of HfO_2-based ferroelectric devices. For instance, in the 45 nm thickness and 10 μm diameter AlScN case at 10 kHz with partial $2P_r = 10$ μC/cm^2, the energy consumption values are close to those reported for HfO_2, showing that partial switching can yield comparable energy use. This highlights that while absolute energy use is generally larger for AlScN, partial polarization operation can mitigate the difference and enable competitive performance.

The higher energy consumption of AlScN compared to HfO_2-based ferroelectrics arises from its intrinsically larger remanent polarization values and the higher coercive fields required to switch them. Larger polarization leads to higher switching currents, and higher coercive fields necessitate higher applied voltages. To enable a more meaningful comparison between the two material systems, we normalized the switching energy by $2P_r$, yielding the parameter E_{PN} / ($2P_r$ × Area). This metric mathematically and physically is same as the effective voltage experienced during unit polarization switching.

Interestingly, the calculated effective voltage is often significantly higher than the externally applied pulse amplitude. This indicates that a portion of the supplied electrical potential energy on the AlScN is dissipated through non-switching processes such as leakage current, defect charging, or thermal losses, rather than being fully converted into ferroelectric switching work. In contrast, for an ideal ferroelectric with negligible leakage, E_{PN} / ($2P_r$ × Area) would mathematically converge to the applied voltage itself. Therefore, the observed deviation from the applied voltage indicates that additional energy is dissipated through non-ideal processes inherent to real devices.

Notably, our results reveal that the effective voltage markedly decreases when partial polarization switching of 10 μC/cm^2 is applied. This observation suggests that the energy consumed per unit polarization is more efficiently utilized for actual dipole switching rather than dissipated through parasitic loss channels. In other words, under limited switching conditions, the supplied energy is concentrated on ferroelectric switching, leading to minimal additional material degradation. This behavior demonstrates that partial polarization operations not only reduce the effective energy cost but also promotes more stable and less destructive switching dynamics, offering a promising route toward energy-efficient and reliable AlScN-based ferroelectric devices.

However, the ideal minimum energy consumption per unit polarization is still limited by the coercive voltage itself. In other words, minimizing the effective energy per unit polarization requires reducing the coercive voltage. This can be achieved by scaling down the ferroelectric thickness. The relatively large AlScN thickness used in this study contributes to the high coercive voltage. Consequently, the elevated effective switching energy observed here. Our previous reports on thinner AlScN films demonstrate that scaling reduces the coercive voltage[1], offering a clear and practical pathway toward more energy-efficient ferroelectric operation. Such optimization directly enhances switching efficiency and will be crucial for achieving energy-competitive ferroelectric operation in future AlScN-based memory technologies.

In addition, the contribution from parasitic capacitance and resistance should also be considered. These parasitic elements can induce additional energy dissipation that does not contribute to ferroelectric switching. Because their absolute values remain nearly constant regardless of device area, their relative influence becomes more pronounced in smaller capacitors. Consequently, the apparent energy consumption per unit area appears larger for smaller devices. As the device size scales down, careful engineering of parasitic capacitance and resistance becomes increasingly important. This trend is observed not only in AlScN but also in HfO_2-based ferroelectrics, as summarized in **Supplementary Table S1**.

In summary, our recalculated comparison indicates that AlScN generally consumes more energy per cycle than HfO_2-based ferroelectrics. At the same time, the observed variations with frequency, electrode diameter, and partial $2P_r$ show that energy use can be modulated through careful design. These results point to the need for continued engineering optimization to balance endurance, polarization density, and energy efficiency in future memory applications.

Supplementary Note 1

Because AlScN is ferroelectric[16] and an inherently piezoelectric material[29], externally applied pressure by a probe tip can induce an internal potential as an intrinsic response. In addition, when the probe tip applies force, the local contact region may deform, and the mechanical boundary conditions of the capacitor can be modified[30, 31]. These combined effects can lead to non-uniform electric field distributions and localized current crowding. Such conditions may further enhance self-induced Joule heating or electron-lattice collisions, which in turn increase unintended phenomena such as dielectric breakdown, electromigration, or other migration phenomena[32]. Consequently, various unexpected degradation effects can trigger abrupt breakdown events. Here, we provide detailed experimental evidence supporting this breakdown behavior.

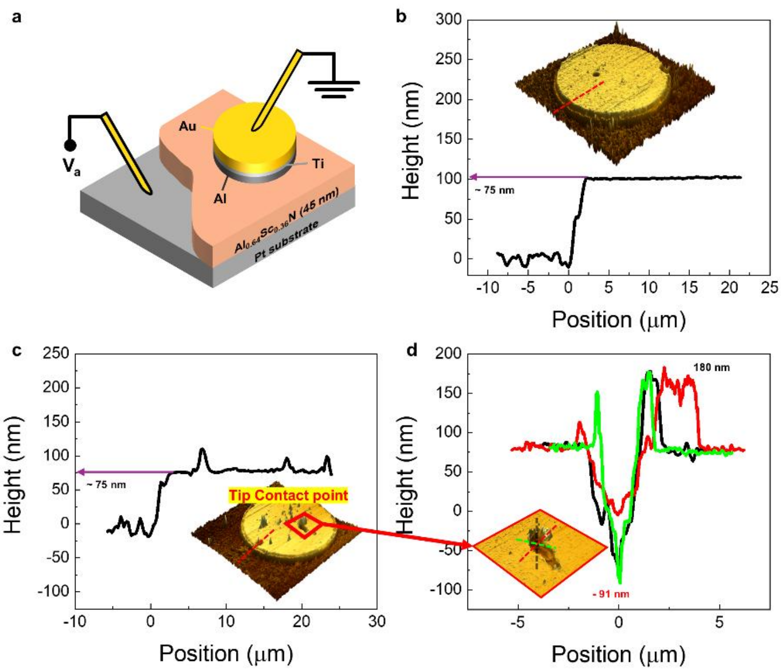

Supplementary Note Figure S1-1 | AFM topography before and after breakdown of a non-via contact capacitor. (a) Schematic illustration of the $Al_{0.64}Sc_{0.36}N$ capacitor with a non-via contact structure and direct tip-contact measurement configuration. **(b)** AFM line-scan profile with inset showing the full 3D topography prior to endurance testing. **(c, d)** AFM line-scan profiles with corresponding 3D topography insets recorded after accidental breakdown, with **(d)** highlighting the tip-contact region. Dashed lines in each inset indicate the AFM scan-line trajectories.

Supplementary Note Figure S1-1 (a) illustrates a capacitor measured without via contacts (direct probe-tip contact to the top electrode), and **Supplementary Note Figure S1-1 (b)** presents an AFM line

scan with a 3D inset obtained prior to any electrical stress (DC-IV, AC-IV, or endurance test). The dashed line indicates the scan trajectory. Before testing, the surface topography is uniform and smooth as shown in **Supplementary Note Figure S1-1 (b)**. By contrast, after an unintentional breakdown, the probe contact site exhibits a crater deformation penetrating into the active AlScN layer, as shown in **Supplementary Note Figure S1-1 (c)** and **S1-1 (d)**. The crater depth reaches up to approximately 91 nm at its deepest point, indicating that the pit extends through the ferroelectric film and reaches the underlying platinum (Pt) bottom electrode. Such penetration explains sudden device failure, as the pit creates a conductive path that produces a direct short. Consequently, the current rapidly rises to the compliance limit, giving the appearance of a maximum current spike. This outcome underscores the risk associated with non-via measurements.

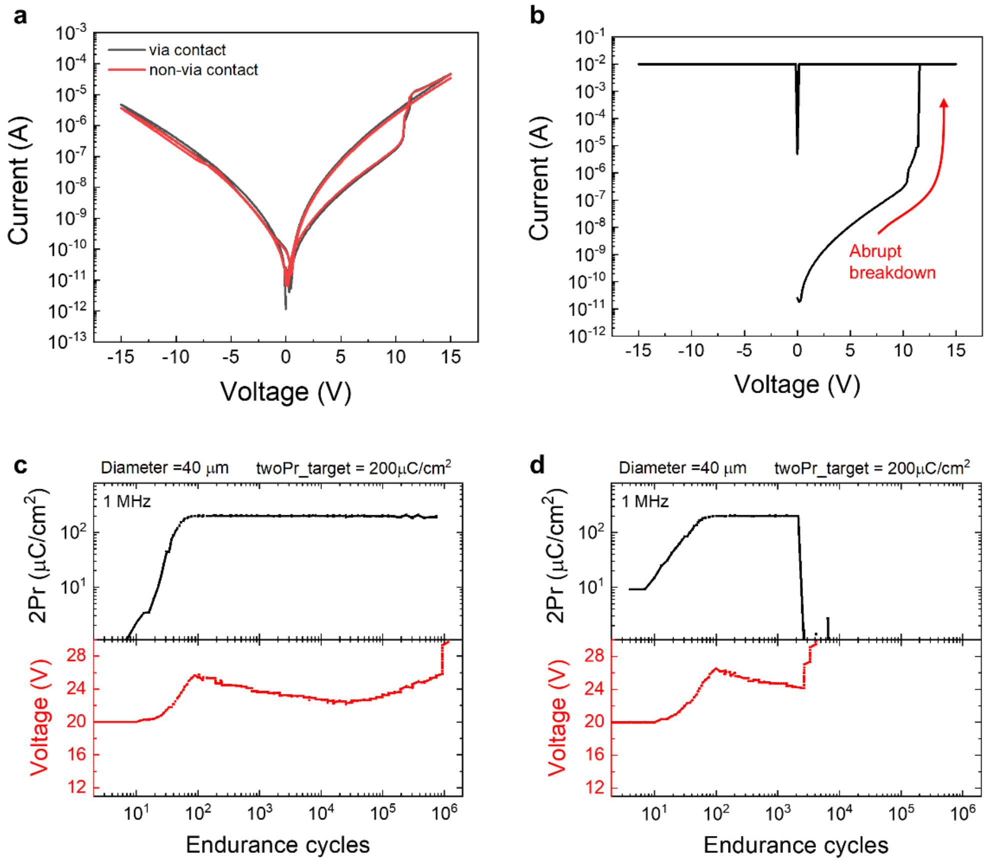

Supplementary Note Figure S1-2 | Electrical comparison between via and non-via contact capacitors. (a) DC-IV of a well contacted device shows stable behavior. The plot includes not only via contact but also non-via contact under gentle touch. **(b)** DC-IV of a non-via contact capacitor under non-gentle touch showing abrupt failure. This is rarely observed with a via contact. **(c)** Endurance of a via contact capacitor under adjusted voltage pulses that keep conventional $2P_r$ close to 200 μC/cm^2.

(d) Endurance of a non via contact capacitor under the same condition of **(c)**.

To further clarify the endurance behavior, additional experiments were performed on capacitors with 40 μm diameter top electrodes under a target preset $2P_r$ of ~200 μC/cm^2 at 1 MHz. The endurance characteristics of via- and non-via configurations are summarized in **Supplementary Note Figure S1-2 (a-d)**.

For non-via contact tests, maintaining "gentle touch" is essential. The probe tip force on the sample must be kept as low as possible such that it touches the electrode lightly without stressing the ferroelectric layer. Under such conditions the AlScN film remains stable and reproducible measurements can be obtained. Indeed, initial DC-IV small voltage range sweeps were used to confirm gentle touch prior to endurance cycling tests for a non-via contact capacitor. Despite this precaution, certain non-via capacitors still failed prematurely around 10^4 cycles, as shown in **Supplementary Note Figure S1-2 (d)**. This behavior is attributed to the inherent fragility of the non-via geometry. Even slight external perturbations can affect the gentle touch configuration and accelerate breakdown.

Moreover, as shown in **Supplementary Note Figure S1-2 (c)** and **(d)**, via contact capacitors and non-via contact capacitors under gentle touch display nearly identical switching in the early stage. This confirms that geometry does not significantly alter the intrinsic electrical properties, provided that gentle contact is maintained in the non-via case. Notably, a via capacitor could still endure up to 10^6 cycles with reliable switching across the full voltage range, as shown in **Supplementary Note Figure S1-2 (c)**. This difference highlights that endurance stability depends not only on the intrinsic characteristics of AlScN but also on the robustness of the contact with probe tip scheme.

These results demonstrate that while the polarization switching response is fundamentally similar, the reproducibility and lifetime are highly sensitive to probing configuration and mechanical loading. The robustness afforded by via contacts, together with careful control of contact force, is therefore essential for achieving reliable long term endurance performance.

To further investigate the post-breakdown state of the non-via contact capacitor, we prepared a device in which the top electrode was selectively removed. The corresponding sample was fabricated with position indicators, as shown in **Supplementary Note Figure S1-3**, to facilitate identification of the location where electrical testing had been performed. For efficient removal of the Al top electrode, we employed a dilute HF solution (5 ml of 2% HF dissolved in 5 ml of deionized water). A device that had experienced abrupt failure during electrical cycling was chosen for this analysis, and AFM imaging was conducted following top electrode removal. **Supplementary Note Figure S1-3 (c)** presents the AFM topography of the breakdown site. The square region highlighted by the white dashed box corresponds

to the original capacitor footprint. The black dashed line is drawn across the region contacted by the probe tip to indicate the line-profile scan trajectory. Line-scan spectroscopy along this black dashed line, as shown in **Supplementary Note Figure S1-3 (d)**, revealed a crater penetrating to a depth of approximately 45 nm. Considering that the Al top electrode was fully etched away, this crater extends nearly to the bottom interface of the ~45 nm-thick AlScN layer, thereby indicating a localized dielectric failure.

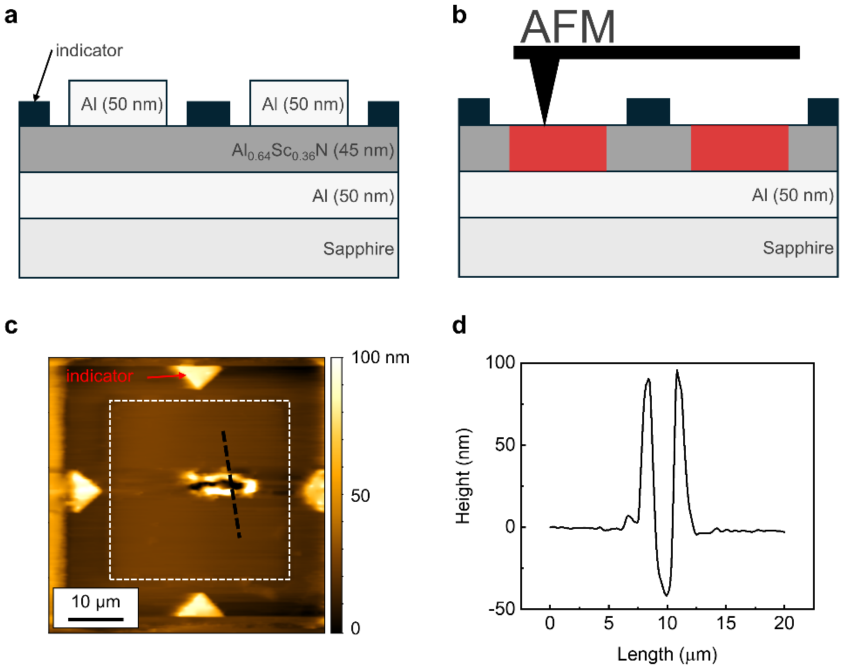

Supplementary Note Figure S1-3 | AFM topography after accidental breakdown of a non-via contact capacitor following top electrode removal. (a, b) Schematic illustration of the non-via contact capacitor before and after electrode etching. The red region in **(b)** denotes the polarization-switched area under applied bias due to the electrical test. **(c, d)** AFM measurements after breakdown, where the dashed black line in **(c)** marks the scan trajectory at the tip-contact location, corresponding to the line profile shown in **(d)**.

These observations indicate that excessive probe loading in non via contact configurations can create sharp local stress and trigger early breakdown. In contrast, via contact structures relieve local stress and improve reliability during endurance testing. This analysis underscores the disadvantage of non via contact devices for long cycling studies and supports the use of via contact designs for robust evaluation of AlScN capacitors.

Besides the benefits discussed above, via structures provide further practical advantages. Via contacts offer a pad area larger than the capacitor itself, which facilitates accurate probe alignment. When the

top electrode diameter is below 15 μm, it is not possible to establish a stable connection using a conventional probe tip in our system. In addition, as noted in our main paper, the contact pad is formed above the region without bottom Al electrode or AlScN piezoelectric layer, allowing it to withstand strong pressure without introducing stress into the AlScN. This ensures stable contact during extended endurance measurements lasting up to fourteen days.

In contrast, non-via structures require exceptionally gentle probe touch to avoid stressing the film. Such delicate contact is often disrupted by vibrations or external perturbations, leading to instability. Despite carefully applying gentle contact, premature breakdown frequently occurred at the early stages of endurance testing. These observations emphasize that, beyond the intrinsic ferroelectric properties, structural considerations are also critical, particularly for materials exhibiting unprecedentedly large $2P_r$ values.

Supplementary Note 2

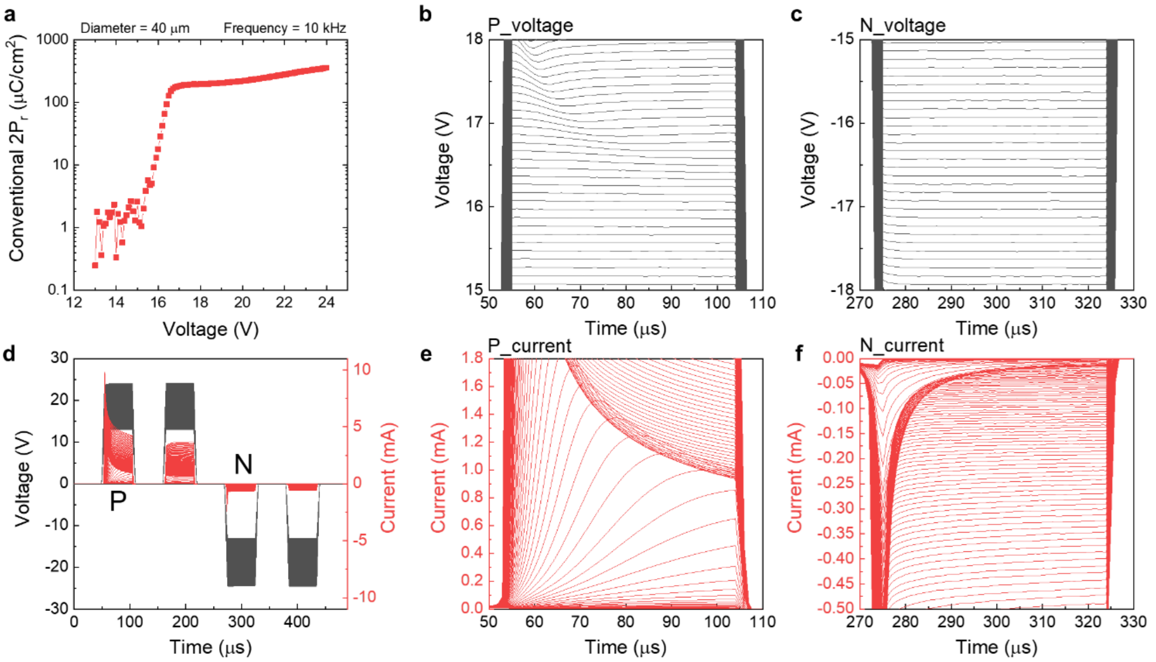

Supplementary Note Figure S2-1 | PUND measurement results at 10 kHz for a capacitor with a 40 μm diameter. (a) illustrates conventional 2P$_r$ behavior as a function of applied voltage. Voltage response during the **(b)** P pulse and **(c)** N pulse. **(d)** PUND measurement results, including both current and voltage responses over the full-time span. Current response during the **(e)** P pulse and **(f)** N pulse.

To deepen the understanding of our AlScN capacitor's partial polarization, we conducted more investigations through the PUND measurements under various conditions. The PUND measurements were conducted systematically by varying the applied voltage pulse in 0.1 V increments and the pulse width. As shown in **Supplementary Note Figure S2-1** and **S2-2,** the observed shift of the current peak to earlier time points, along with its increasing sharpness and magnitude, indicates that such behavior occurs during the polarization switching process. This behavior indicates that domain nucleation and growth occur progressively, with only a fraction of ferroelectric domains switching per pulse instead of undergoing instantaneous collective reversal.

These results are similar to prior studies[33-37]. Their studies demonstrated that the kinetics of ferroelectric switching are strongly influenced by the external field amplitude, pulse duration, and pre-existing domain structures. Guido et al.[36] showed that in AlScN capacitors, polarization switching occurs through domain nucleation and wall motion, with higher electric fields accelerating switching. Yazawa et al.[37] also reported that in wurtzite-structured ferroelectrics, increasing the electric field leads to sharper, larger, and faster current peaks during polarization switching.

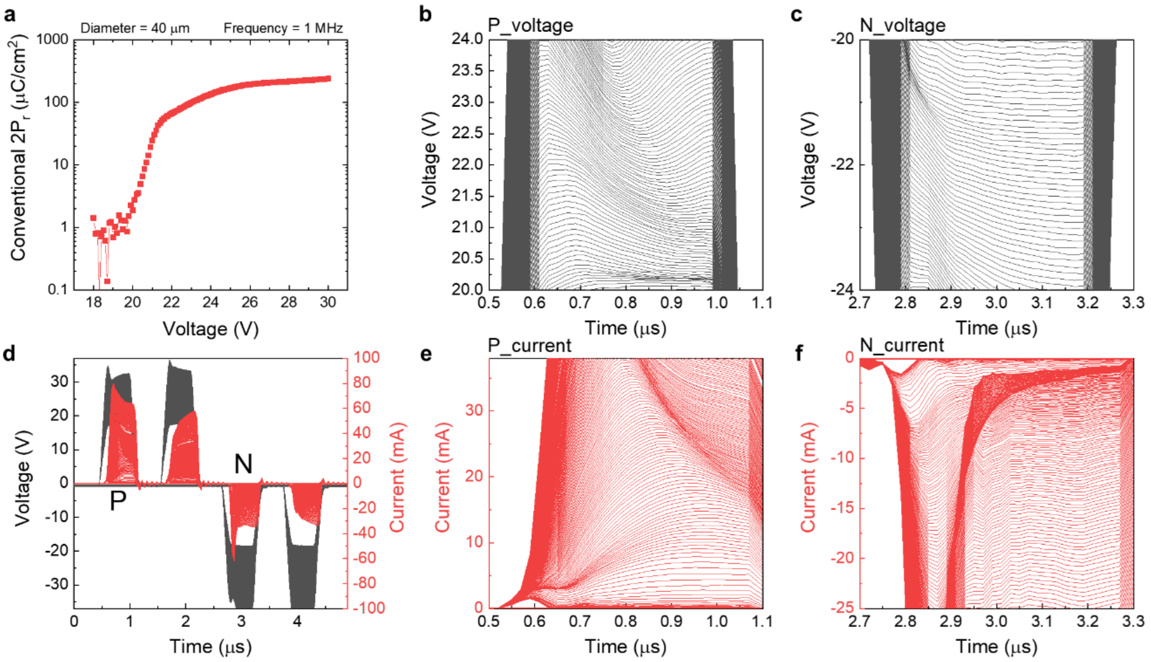

Supplementary Note Figure S2-2 | PUND measurement results at 1 MHz for a capacitor with a 40 µm diameter. (a) illustrates conventional $2P_r$ behavior as a function of applied voltage. Voltage response during the **(b)** P pulse and **(c)** N pulse. **(d)** PUND measurement results, including both current and voltage responses over the full-time span. Current response during the **(e)** P pulse and **(f)** N pulse.

The increase in the current peak height observed in our measurements provides further evidence of an enhanced switching efficiency as the applied voltage increases. This trend suggests that a greater proportion of ferroelectric domains participate in the switching process at higher fields, leading to an amplified current response. The increase in peak current is likely a consequence of the progressive reduction in activation energy barriers for domain nucleation, as well as the cumulative contribution of switched dipoles. Such behavior is similar to the previous report[34].

These results can also provide evidence of negative conventional $2P_r$ during the early stages of the endurance test that appeared in **Figure 3c**. The origin of this apparent anomaly lies in the switching peak emerging near the end of the P pulse. As a result, the polarization-induced resistance state is effectively established by the time the U pulse is applied. The U pulse therefore propagates through a more conductive configuration, producing an enhanced leakage-assisted current. Consequently, the integrated charge from the U pulse surpasses that of the P pulse, yielding a negative value of P–U. This phenomenon reflects the interplay between polarization-dependent leakage and switching kinetics, both of which distort the conventional $2P_r$ extracted in the partial polarization regime.

Supplementary Note 3

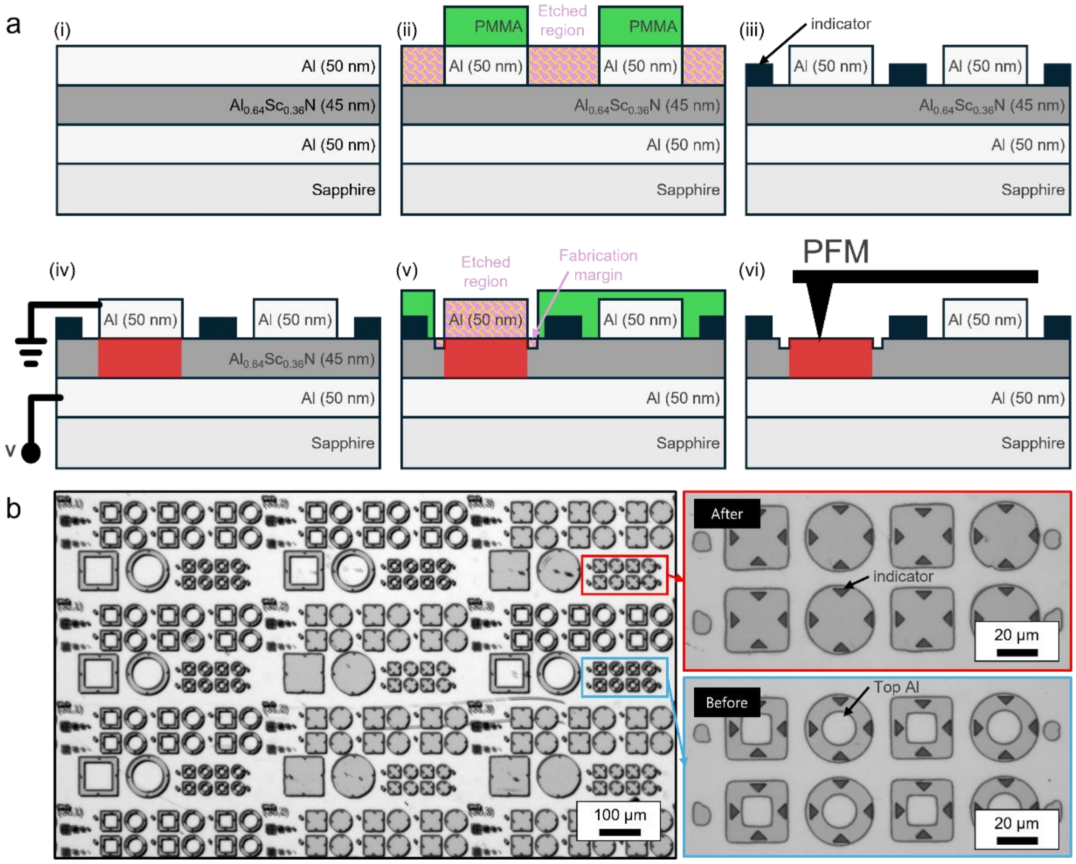

Supplementary Note Figure S3-1 | Preparation of the PFM test sample. (a) Schematics of the sample preparation process. **(b)** Optical microscopy (OM) images of the AlScN PFM sample. The large panel on the left shows the entire array, and the two small panels on the right show magnified views.

For piezoresponse force microscopy (PFM) measurements the sample preparation is illustrated in **Supplementary Note Figure S3-1**. In **Supplementary Note Figure S3-1a**, **(i)** Al/AlScN/Al were deposited in situ on a 6-inch sapphire wafer, with details in the methods section in our paper. **(ii)** The top Al was wet etched in a dilute HF solution prepared by mixing 5 ml of 2 % HF with 5 ml deionized water, followed by electron beam lithography (EBL). The patterned PMMA resist served as a mask for wet etching, and the pink region denotes the etched area. **(iii)** An Au/Ti indicator was patterned by an EBL process and deposited via electron beam evaporation. **(iv)** Electrical measurements were subsequently conducted to prepare various switched states of the AlScN layer. We applied the voltage on the bottom Al electrode and grounded the top Al. The red box indicates selectively the switched sites

of AlScN. **(v)** We opened only the switched sites by the EBL process and PMMA development. Subsequently, we repeated the dilute HF etch in step **(ii)** to remove the top Al and expose AlScN for the PFM test. In practice, PMMA does not form perfectly sharp edges. To prevent residual top Al metal from remaining at the capacitor edge, we left a small margin. As a result, a narrow area next to the AlScN region marked in pink was also etched where the PMMA did not fully cover. This fact supports the PFM analysis presented in **Supplementary Note Figures S3-3 to S3-5**. **(vi)** Consequently, PFM was performed. The PFM analysis was carried out in an Asylum MFP-3D AFM with an Nanosensors PPP-EFM tip of 75 kHz resonant frequency and 2.8N/m spring constant.

In **Supplementary Note Figure S3-1b** the large OM image on the left presents the full patterned array. The red box shows the state after step **(v)**, in **Supplementary Note Figure S3-1a**, used to prepare for the PFM test. The blue box shows the state after step **(ii)**, in **Supplementary Note Figure S3-1a**, used for the electrical tests. The right panels provide magnified views labeled "Before" and "After". "Before" shows the top Al electrode still present. "After" shows selective removal of the top Al, which exposes AlScN at the intended locations. The indicator is visible in both states and serves as a reference marker that guides us back to the exact switched region and allows precise PFM tip placement.

Supplementary Note Figure S3-2 provides the PFM on AlScN that was switched to a partial polarization state with preset $2P_r = 100\ \mu C/cm^2$. **Supplementary Note Figure S3-2a** shows the AFM topography where partially switched AlScN is present. Particularly, a triangular metal indicator marks the site where pulses were applied. **Supplementary Note Figure S3-2b** and **c** display amplitude and phase, respectively, from the same area. To perform the PFM measurement, an electrical pre-poling process was conducted before imaging. Since our study focuses on polarization defined with respect to negative $2P_r$, the film was first initialized to the M-polar state by applying two positive voltage pulses to the bottom electrode exceeding the coercive voltage. Although a single pulse can align most dipoles, a second pulse was applied to ensure complete switching and stabilization of the M-polar state. Subsequently, two negative voltage pulses were applied to induce partial polarization. The extent of partial polarization of $100\ \mu C/cm^2$ was calculated using the conventional PUND method. As discussed in **Supplementary Figure S6**, applying two pulses causes two switching events, leading to a larger polarization change than a single-pulse operation. While the targeted switching level was set to $2P_r \approx 100\ \mu C/cm^2$, the actual polarization change reached approximately $125\ \mu C/cm^2$.

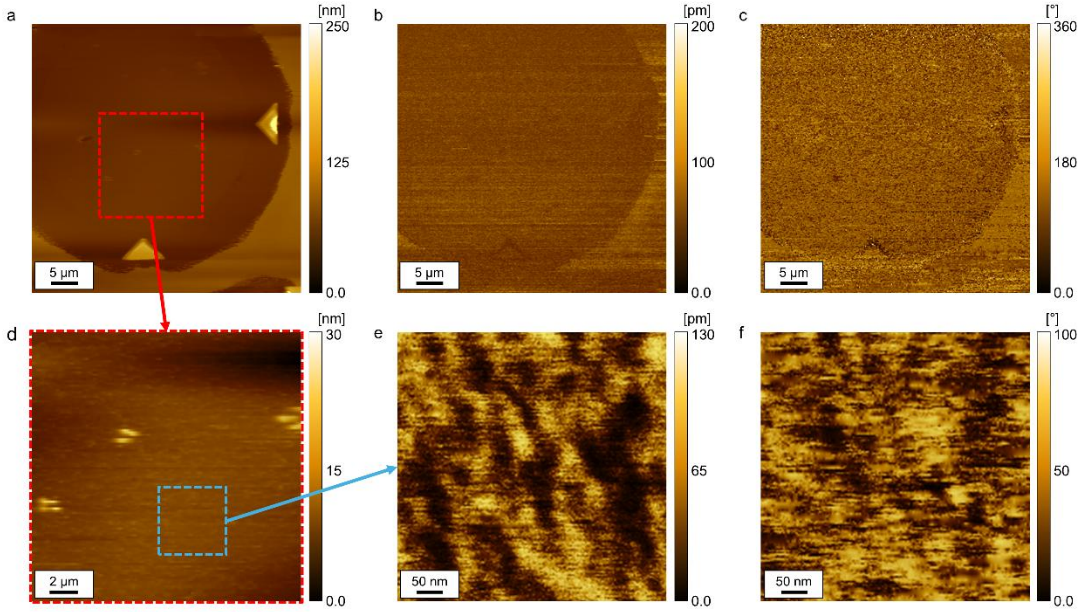

Supplementary Note Figure S3-2 | PFM Test results of preset $2P_r$ = 100 μC/cm². PFM test results for **(a)** height topography, **(b)** amplitude and **(c)** phase images which are prepared for the 100 μC/cm² of $2P_r$. **(d)** Enlarged view of the tested region marked in the overview. The highest magnified images with 1×1 μm² region of **(e)** amplitude and **(f)** phase.

The amplitude and phase contrasts in **Supplementary Note Figure S3-2** are not significantly different from those of pristine AlScN, which predominantly exhibits N-polar orientation. This similarity makes it difficult to distinguish the partially switched region from the pristine area, in **Supplementary Note Figures S3-2b** and **c**. Nevertheless, the switched polarization still remains below the saturation level of AlScN. As shown in **Figure 1e**, full polarization in AlScN reaches approximately 200 μC/cm², confirming that the state observed in **Supplementary Note Figure S3-2** corresponds to partial polarization. However, it is still difficult to observe evidence of partial polarization in **Supplementary Note Figure S3-2 a–c** because of the relatively large scale.

Supplementary Note Figures S3-2 from **d** to **f** provide enlarged views bridging the overview in **Supplementary Figure S3-2a** and nanoscale scans. The 500 × 500 nm² high-resolution amplitude and phase maps (**Supplementary Note Figures S3-2e** and **f**) reveal clear evidence of partial polarization. The amplitude contrast is spatially non-uniform, while the phase exhibits two distinct states separated by ~180°, indicating only partially switched dipoles are present within the switched region. This

coexistence of switched and unswitched domains directly proves incomplete switching in AlScN. These findings are consistent with earlier studies[38-40] and confirm that stable intermediate polarization states can be reliably achieved in nitride ferroelectrics.

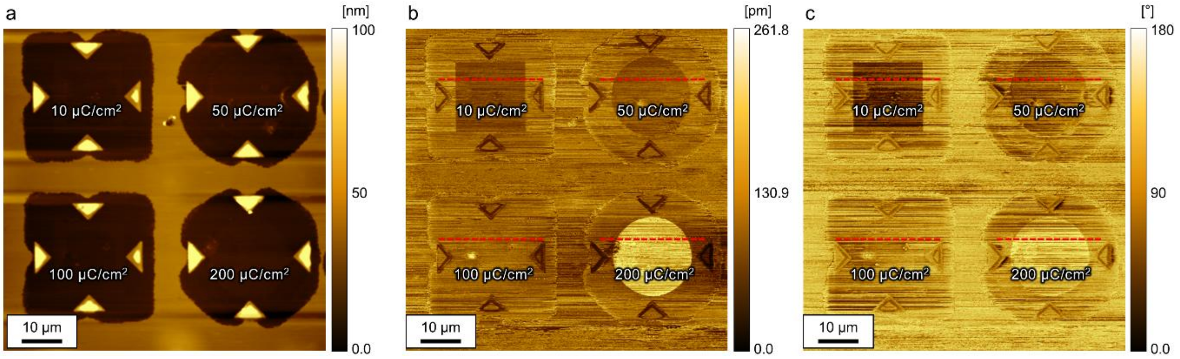

Supplementary Note Figure S3-3 | PFM images of AlScN under various preset 2P_r conditions. The images consist of **(a)** topography, **(b)** amplitude, and **(c)** phase maps acquired simultaneously in a single scan. The regions indicated by triangular metal markers correspond to areas where voltage pulses were applied to induce switching. The extent of preset polarization for each region is labeled directly in the images. The red dashed lines in the amplitude and phase images indicate the locations of line profiles, which are analyzed in **Supplementary Note Figure S3-4**.

Supplementary Note Figure S3-3 presents a comprehensive set of PFM images obtained under different preset 2P_r conditions ranging from 10 to 200 μC/cm^2. These images were obtained by scanning a wide area (80×80 μm^2) in a single acquisition, minimizing errors caused by different measurements. The scan was performed at a very slow rate of 0.1 Hz to further reduce measurement artifacts. In **Supplementary Note Figure S3-3a** shows no significant differences between switched and unswitched regions, confirming that the HF etching process was performed uniformly.

In contrast, the amplitude and phase responses shown in **Supplementary Note Figures S3-3b** and **c** exhibit a clear dependence on the preset 2P_r. As the preset value increases, the switched regions appear progressively brighter, and the contrast with unswitched areas becomes more distinct. As discussed in **Supplementary Note Figure S3-2**, the region preset to 2P_r = 100 μC/cm^2, which is close to the pristine N-polar predominant AlScN state, shows only subtle contrast differences, making it difficult to distinguish the switched area. However, when the preset is increased to 200 μC/cm^2, both amplitude

and phase images display a pronounced bright region, clearly distinguishing the switched domains from the surrounding matrix.

The topography in **Supplementary Note Figure S3-3a** shows no noticeable morphological difference between switched and unswitched regions, confirming that the applied bias and etch process does not cause significant surface deformation or damage. In contrast, the amplitude and phase represent the overall response within the scanned area, reflecting how much of the region was switched by the preset pulse. As the preset $2P_r$ increases, both amplitude and phase contrast become stronger, indicating a gradual and cumulative change in the overall polarization state. These results confirm that the polarization in AlScN can be continuously adjusted by controlling the preset $2P_r$, allowing fine control over the average polarization density.

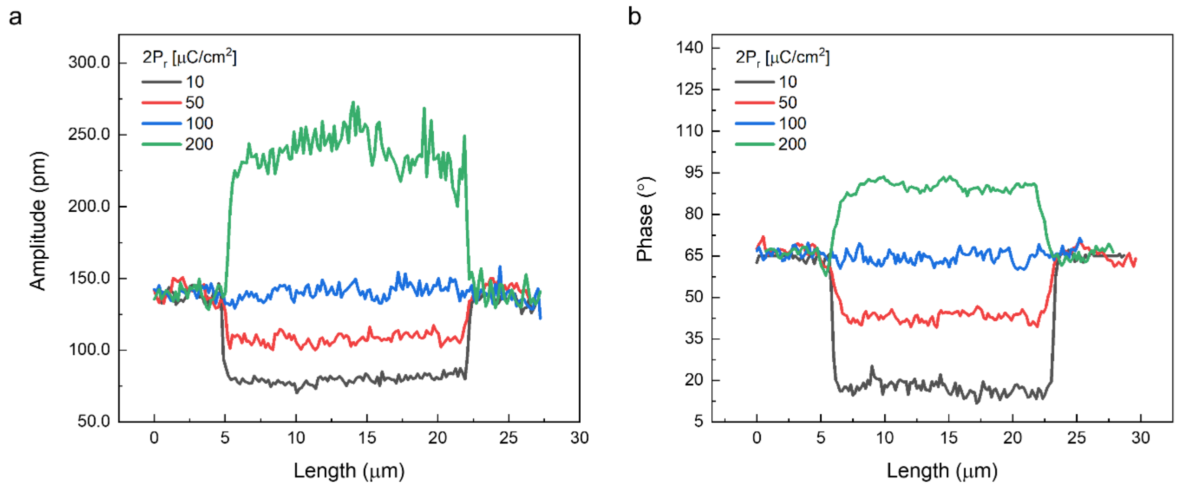

Supplementary Note Figure S3-4 | PFM amplitude and phase line profiles under various preset $2P_r$ conditions. (a) Amplitude line profiles and **(b)** phase line profiles are extracted along the red dashed lines indicated in **Supplementary Note Figure S3-3**. Each profile corresponds to different preset $2P_r$ values (10, 50, 100, and 200 μC/cm^2).

In **Supplementary Note Figure S3-4**, PFM line profiles were compared under different preset $2P_r$ conditions to highlight the effect of partial polarization. Both amplitude (**Supplementary Note Figure S3-4a)** and phase (**Supplementary Note Figure S3-4b**) profiles clearly distinguish between the switched region (from 5 to 23 μm along the length) and the unswitched region (other regions). Notably, the amplitude and phase increase systematically with larger preset $2P_r$, indicating that the extent of domain reversal scales with the applied polarization. In summary, these observations confirm that the

amplitude and phase response reflects the progressive evolution of partially switched domain fraction with increasing preset $2P_r$. These results support again the nanoscale evidence of controlled ferroelectric in AlScN films.

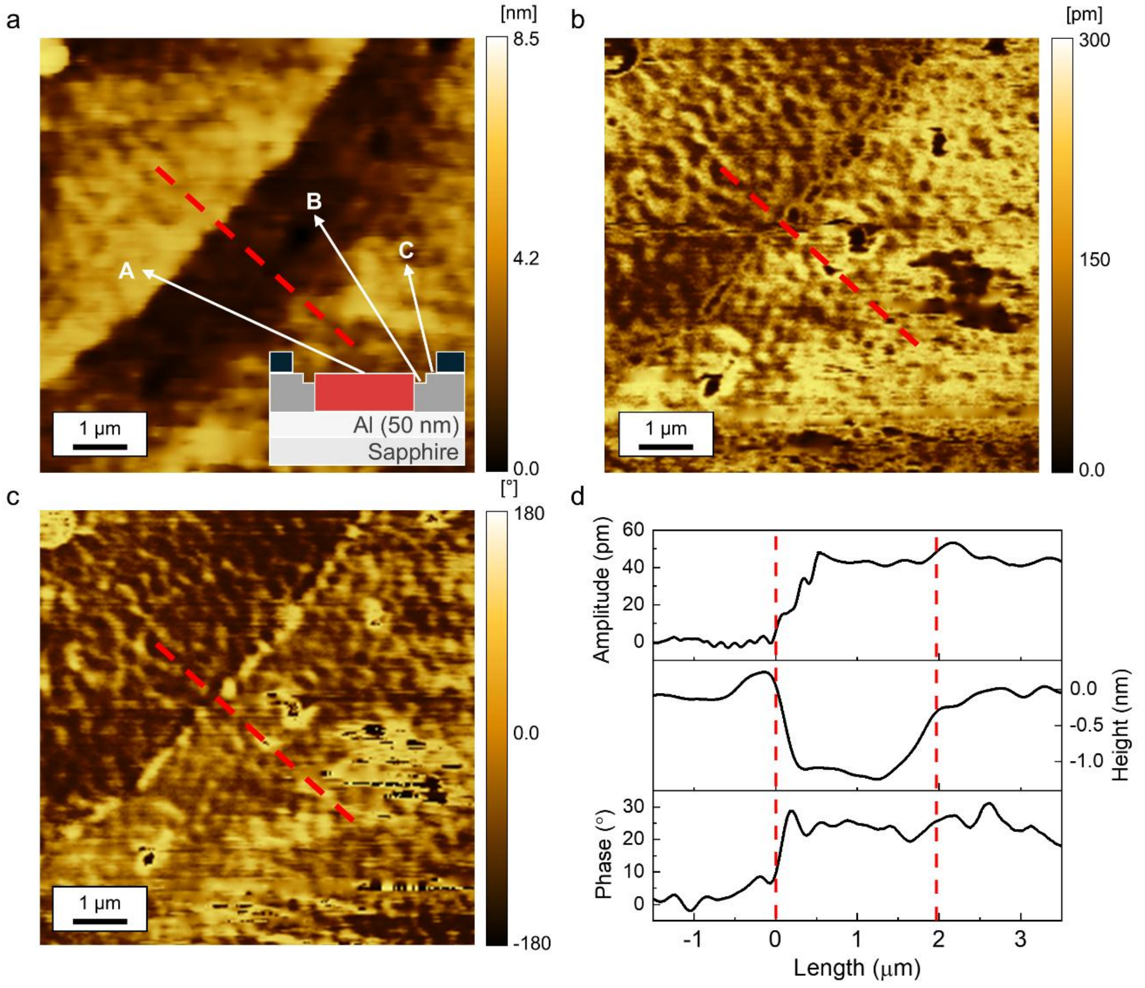

Supplementary Note Figure S3-5 | PFM images and line profiles across the switched boundary of preset $2P_r$ = 50 μC/cm^2. (a–c) present the PFM topography, amplitude, and phase images of the same region**.** The inset in **(a)** illustrates the schematic structure of the sample, where arrows indicate the correspondence between the schematic and the PFM images. The red dashed lines mark the positions along which the line profiles were extracted. **(d)** shows the resulting line profiles of height, amplitude and phase across the domain boundary, clearly distinguishing the switched and unswitched regions. The red dashed line separates regions A, B, and C. The left part corresponds to region A, the middle to region B, and the right to region C.

In **Supplementary Note Figure S3-5**, we further examined whether HF etching influences the amplitude and phase responses, which could otherwise complicate interpretation. Regions A, B, and C in **Supplementary Note Figure S3-5a** share the AlScN surface but differ in condition. Region A is electrically switched, region B is unswitched but partially etched due to fabrication margin in step **(v)** of **Supplementary Note Figure S3-1a**, and region C is unswitched and fully protected by PMMA, remaining close to the as-grown state. The white arrow in the inset of **Supplementary Note Figure S3-5a** highlights the etched margin in region B, confirmed by the height profile. **Supplementary Note Figure S3-5d** shows region B is ~1 nm lower than A and C. While such height variations could affect PFM detection, optimized measurement conditions eliminate this influence. The amplitude and phase responses depend only on switching state, not surface topography, confirming that the observed contrast arises from ferroelectric polarization rather than etching artifacts.

To complement these experimental results, we further introduce a theoretical framework describing incomplete domain switching in AlScN. Recent studies on wurtzite ferroelectrics have established that at higher Sc content, the switching pathway transitions from a collective cation-anion sublattice displacement to a sequential inversion of tetrahedral units via intermediate nonpolar structures[41-43]. This mechanism lowers the effective switching barrier and enables the stabilization of metastable intermediate polarization states. As a result, this theoretical background demonstrates that partial polarization is theoretically feasible. These intermediate states can remain stable over repeated cycling, allowing the system to sustain non-fully switched configurations without relaxation. Under such partial polarization, the effective electric field acting on each domain is reduced, suppressing the nucleation rate of reverse domains. Consequently, the per-area probability that a critical nucleus will develop, and collapse diminishes, lowering the instantaneous hazard for breakdown and extending the time to failure[44]. Our electrical data, showing extended endurance beyond 10^{10} cycles under partial polarization ($2P_r \approx 10$ - $100\ \mu C/cm^2$) compared to ~10^8 cycles for full polarization reversal ($2P_r \approx 200\ \mu C/cm^2$), are consistent with this theoretical model.

The observed PFM features are consistent with a nucleation limited and defect pinned switching regime in wurtzite AlScN[36, 45-47]. Under electric fields below the coercive voltage, polarization switching proceeds by nucleation at easy sites and short-range domain wall propagation rather than a collective 180° flip[48, 49]. When a wall encounters a pinning center, bound charge and the associated internal field accumulate at the local wall segment. At a charged wall segment the internal field points opposite to the

external drive. The effective driving field on the wall is therefore reduced. Extra work is then required to propagate the wall. In our films (~ 50 nm) incomplete screening at the electrodes and at interfacial layers further strengthens this effect[50-57]. These results show a metastable domain pattern that explains the mixed amplitude contrast and the distinct phase response. The phase images show pinned domains and limited wall motion in partially switched AlScN, which persist until higher fields are applied.

In summary, our microscopic PFM measurements and theoretical support provide a framework for understanding enhanced endurance in AlScN. The stabilization of partial polarization states by domain wall pinning and intermediate structural states help the material to avoid the extreme electric field stress required for full switching. Consequently, fatigue and electrical stress are mitigated, Joule heating is reduced, and the overall reliability of the capacitor is significantly improved. These insights not only strengthen our interpretation but also highlight a general principle by which partial polarization control can be used to extend the operational lifetime of wurtzite AlScN ferroelectrics.

Supplementary Note 4

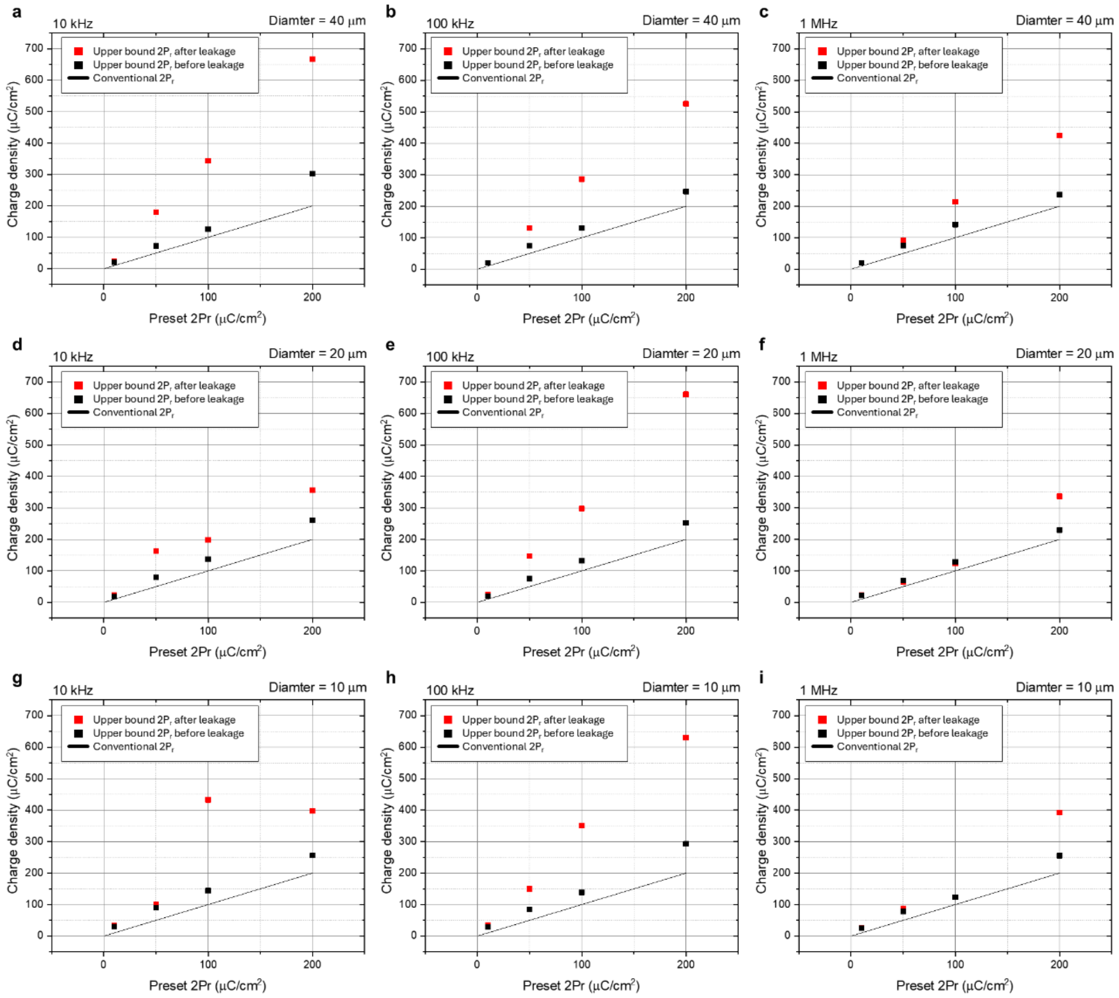

Supplementary Note Figure S4-1 | Relationships between preset 2P$_r$ and various 2P$_r$ parameters under different conditions. Each graph contains an endurance test frequency and the area of AlScN capacitors. The line on the graph represents the conventional 2P$_r$, which is the same value as the preset 2P$_r$ due to the operation of a well-maintaining algorithm.

The conventional PUND method is not appropriate to determine partial polarization. Firstly, there is a possibility of underestimation. Conventional 2P$_r$ is calculated using the P-U (subtracting the integrated charge U from P) and the N-D method, both divided by the capacitor area (details in **Supplementary Figure S3**). The first pulse response consists of both leakage (Q_{leak}) and switching (Q_{2Pr}) charges (P or N = Q_{leak} + Q_{2Pr}), while the second pulse response ideally consists of only (U or D = Q_{leak}). Thus, subtracting the second pulse response from the first (Q_{leak} + Q_{2Pr} - Q_{leak}) theoretically yields Q_{2Pr}. However, the conventional 2P$_r$ derived from these methods is not equivalent to partially switched intrinsic 2P$_r$. When the applied voltage is below the coercive voltage, not all polarization domains fully switch during the initial pulse (P or N = Q_{leak} + $Q_{2Pr\_partial}$; $Q_{2Pr\_partial}$ = intrinsic 2P$_r$). This incomplete

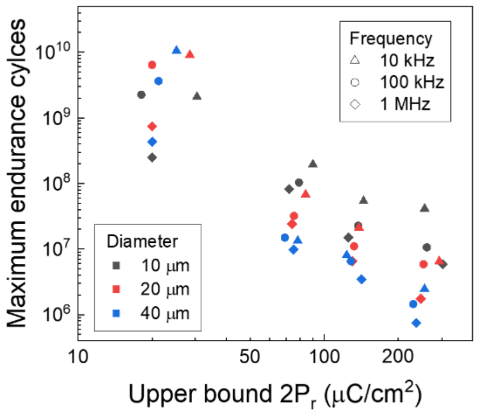

Supplementary Note Figure S4-2 | The relationship between endurance and upper-bound $2P_r$ before leakage subtraction.

switching leaves residual polarization in the material. When the subsequent pulse is applied, this residual polarization contributes to the charge response (U or D = Q_{leak} + $Q_{2Pr\_residual}$). Consequently, the N-D/A calculation underestimates the intrinsic partially switched polarization, resulting in (Q_{leak} + $Q_{2Pr\_partial}$) - (Q_{leak} + $Q_{2Pr\_residual}$) = ($Q_{2Pr\_partial}$ - $Q_{2Pr\_residual}$) instead of the true intrinsic $2P_r$. Unfortunately, no precise methodology exists to obtain partially switched intrinsic $2P_r$. Therefore, we adopted an alternative approach by employing P/area (N/area) values instead of P-U/area (N-D/area) in **Figures 3c** and **3d**. While not perfect, this method was chosen to resolve the limitations of existing techniques and to provide the most reliable estimation possible under the given constraints.

These parasitic effects become increasingly significant at high applied voltages but are largely negligible when the voltage remains low. As demonstrated in **Figure 3c**, where the preset $2P_r$ is 10 μC/cm^2, these interfering factors have a negligible influence due to the low amplitude of the applied voltage. Consequently, the intrinsic $2P_r$ value is expected to be between 10 and 34.25 μC/cm^2. Since the effects of leakage and other parasitic elements are insignificant under such conditions, the upper bound of 34.25 μC/cm^2 serves as a reliable approximation for estimating the partially switched intrinsic $2P_r$.

Expanding upon this approach, we further examined the conditions necessary for achieving fully switched intrinsic $2P_r$. Complete polarization switching requires the application of a sufficiently high voltage, which inherently introduces some degree of overestimation. However, before the onset of leakage or material degradation, the capacitor can be driven at relatively low voltages where such overestimation is significantly reduced. Under these conditions, N values remain close to the full $2P_r$ value of 200 μC/cm^2. This suggests that our AlScN capacitor exhibits minimal leakage currents and a low density of defects, contributing to reliable electrical performance. The limited influence of parasitic effects supports the validity of the estimation approach used in this study.

Supplementary Note Figure S4-1 provides an analysis of the upper-bound $2P_r$ before and after leakage, utilizing the method outlined in **Figures 3c** and **3d** under various conditions. As frequency increases, the difference between upper-bound $2P_r$ and conventional $2P_r$ diminishes. This is because ferroelectric switching occurs at an inherently high speed, allowing a rapid response at high frequencies. In contrast, leakage effects contribute minimally to overestimation at high frequencies due to the extremely short pulse width time. When the preset $2P_r$ is low, the difference becomes even smaller, which is attributed to the reduced contribution of leakage-induced overestimation at lower polarization states due to relatively low applied voltage. This trend is consistently observed across different diameters.

Supplementary Note Figure S4-2 shows the relationship between endurance and upper-bound $2P_r$ before leakage.

Supplementary Note 5

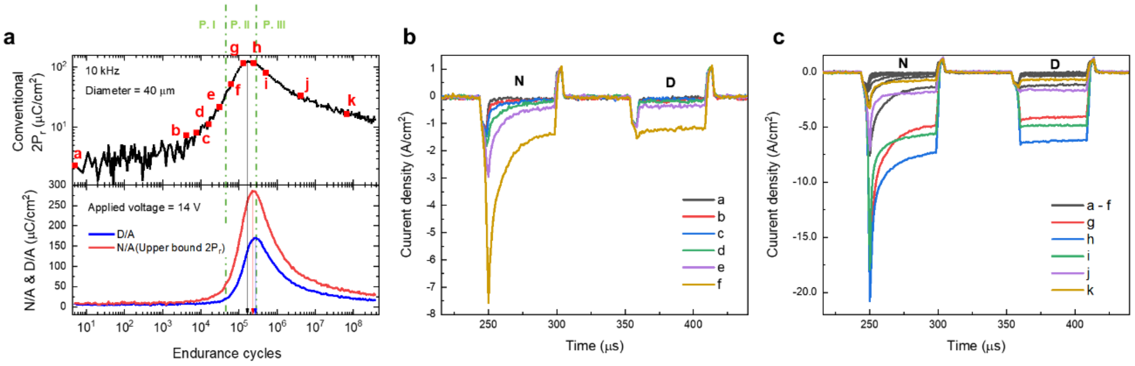

Supplementary Note Figure S5-1 | The endurance test results under a fixed applied voltage of 14 V, the evolution of polarization and current response across different endurance cycles. (a) The top panel shows the evolution of conventional $2P_r$ as a function of endurance cycles, with key transition points marked in green. The bottom panel compares the N/A (upper bound $2P_r$) and D/A current densities. **(b)** and **(c)** exhibit the N and D current responses as a function of time in the partial wake up followed by leakage and degradation phase respectively at different endurance cycle moments.

Supplementary Note 5 provides a clear analysis of how voltage affects ferroelectric switching, dividing it into distinct phases. **Supplementary Note Figure S5-1a** shows a case where a consistent 14 V is applied. The conventional $2P_r$ is shown on a logarithmic y-axis, while N/A (upper bound $2P_r$) and D/A are on a linear scale.

The N peak is mainly attributed to ferroelectric polarization switching. However, since the applied voltage is below the V_C, the switching does not fully reflect the inherent total spontaneous polarization. Additionally, because the material has not fully woken up, the measured polarization does not intrinsically represent the maximum possible switching. As a result, some residual ferroelectric response appears in the D pulse. The current level is also affected by various factors, but the most dominant factors are the charge accumulation due to the paraelectric properties and the leakage current passing through the insulator. Overall, the N current response mainly includes ferroelectric switching and leakage, while the D current response contains a smaller portion of ferroelectric switching along with leakage.

Through the changes in N/A and D/A, three phases can be distinguished. Phase (P.) P.I, P. II, and P. III, as separated by the green dashed line in **Supplementary Note Figure S5-1a**. P. I shows little change in both N/A and D/A, P. II exhibits an exponential increase in N/A and D/A, and P. III marks the beginning of degradation, starting from peak point of D/A. As shown in the traces labeled a-e in **Supplementary Note Figure S5-1b**, P. I is characterized by a steady increase only in the peak of the N

current response, indicating the wake-up process of AlScN. Since the applied voltage of 14 V is much lower than the V_C of 17 V, the wake-up progresses very slowly, this condition is referred to as partial wake-up. This term describes the gradual activation of ferroelectric switching, where polarization begins to change but has not yet to reach its full potential. The graphs labeled a-e in **Supplementary Note Figure S5-1b** also shows that the D pulse remains mostly unchanged, suggesting that leakage does not increase significantly and that the material is still in a partial wake-up stage.

A transition occurs from P. I to P. II, particularly in the traces labeled e to f in **Supplementary Note Figure S5-1b**, where an increase in the D current response indicates the beginning of leakage. In the graphs labeled f-h of **Supplementary Note Figure S5-1c**, not only partial wake-up but also leakage continues to grow during P. II. The transition from P. II to P. III seen in the traces h-k in **Supplementary Note Figure S5-1c** indicates the degradation phase, where both the N and D peak current responses start to decrease due to the material's fatigue phase.

In addition, as seen in **Supplementary Note Figure S5-1a**, the peaks of conventional $2P_r$ appear first, followed by the peak of N/A and then D/A. This is because the rates of partial wake-up and leakage increase at different speeds. Partial wake-up happens more quickly and grows faster than leakage during P. I. Whereas in P. II, leakage starts increasing more quickly, indicating a shift in the dominant process. Understanding this interaction between wake-up and leakage provides important insights into the long-term stability and reliability of ferroelectric materials.

Supplementary Note Figure S5-2 shows the case where the applied voltage is fixed at 16 V. Since this voltage is close to V_C, a relatively faster wake-up process was observed. Similar to **Supplementary Note Figure S5-1**, the region where conventional $2P_r$ increases is also observed in P. I. In **Supplementary Note Figure S5-2b**, the traces labeled a-e show no noticeable increase in the D current response, indicating that leakage remains suppressed while partial wake-up is progresses. During P. I, unlike in **Supplementary Note Figure S5-1**, the conventional $2P_r$ approaches nearly 200 $\mu C/cm^2$. This value represents full spontaneous polarization while leakage remains minimal. This suggests a mitigated wake-up process, providing insights into optimizing wake-up behavior. Furthermore, unlike in **Supplementary Note Figure S5-1**, in P. II, the conventional $2P_r$ exhibits a minimal increase and remains stable. In this phase, N/A and D/A increase at a very slow rate. As observed in the traces labeled f-i in **Supplementary Note Figure S5-2c**, the rates of increase for D and N show nearly the same trend, which suggests the system is in a stable phase. Following this stable phase, the system turns into P. III, characterized by an exponential increase of D/A. Eventually, after reaching the peak of D/A, both the N and D peak current responses start to decrease, as shown in **Supplementary Note Figure S5-2c** and **S5-2d**.

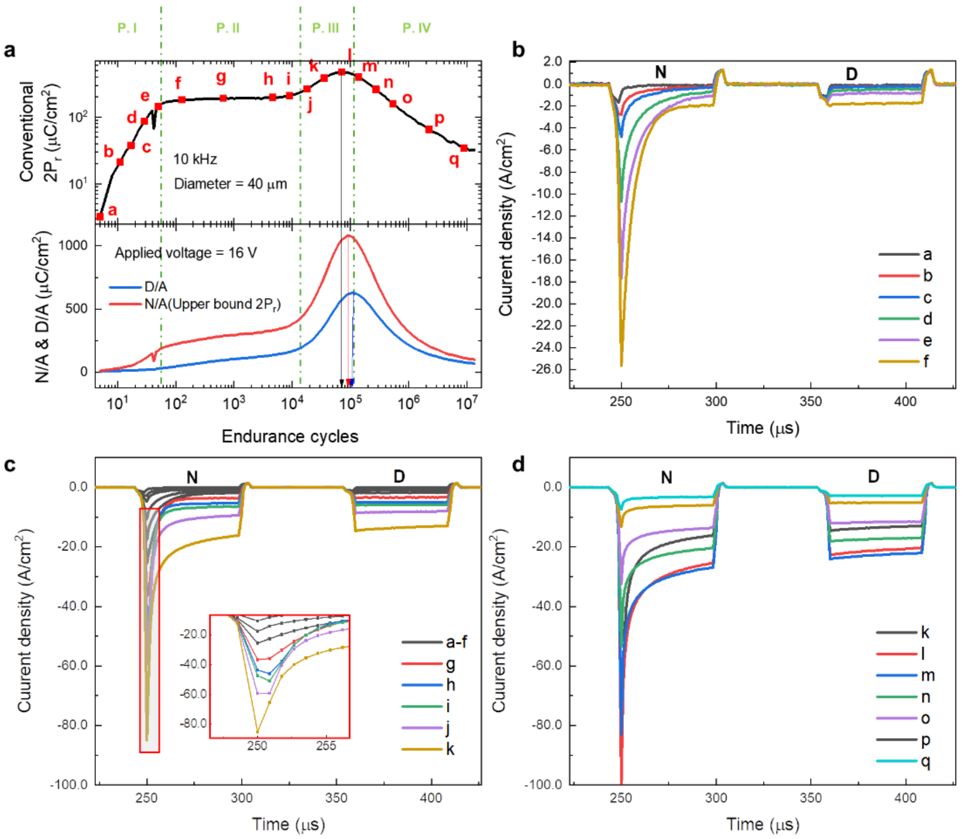

Supplementary Note Figure S5-2 | The endurance test results under a fixed applied voltage of 16 V, the evolution of polarization and current response across different endurance cycles. (a) The top panel shows the evolution of conventional $2P_r$ as a function of endurance cycles, with key transition points marked in green. The bottom panel compares the N/A (upper bound $2P_r$) and D/A current densities. **(b-d)** exhibit the N and D current responses as a function of time in the partial wake up, stable and degradation phase respectively at different endurance cycle moments. The inset in **(c)** is a magnified image at the peak of the N current density response.

In **Supplementary Note Figure S5-3**, a fixed voltage of 18 V, which is higher than V_C, is applied. In this case, no partial wake-up process is observed. As a result, the stable phase that appeared in P. II of **Supplementary Note Figure S5-2** directly emerges in P. I of **Supplementary Note Figure S5-3a**. As shown in **Supplementary Note Figure S5-3b**, traces a–e show a stable state where both the N and D peak current responses continuously increase at the same rate. This stable state is followed by P. II and P. III, where the responses increase exponentially and then decrease.

When a low voltage is applied, the system does not reach its maximum potential value of 200 μC/cm^2. Instead, it enters a phase where leakage increases without the system stabilizing. Conversely, applying a high voltage causes the system to transition directly into a stable state. However, as seen in

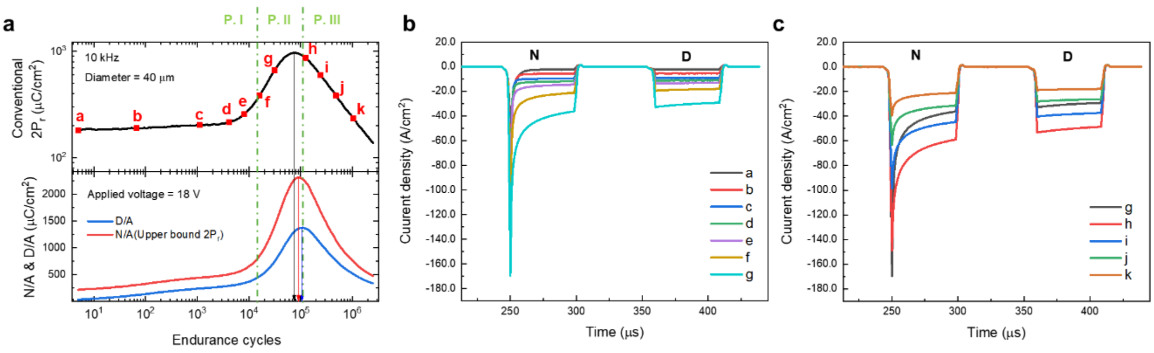

Supplementary Note Figure S5-3 | The endurance test results under a fixed applied voltage of 18 V, the evolution of polarization and current response across different endurance cycles. (a) The top panel shows the evolution of conventional $2P_r$ as a function of endurance cycles, with key transition points marked in green. The bottom panel compares the N/A (upper bound $2P_r$) and D/A current densities. **(b-c)** exhibit the N and D current responses as a function of time in the stable and degradation phase respectively at different endurance cycle moments.

Supplementary Note Figure S5-3a, the D/A peak is approximately ten times higher than that of **Supplementary Note Figure S5-1a**. This indicates a significant increase in leakage current due to the high applied voltage. This elevated leakage induces current stress, resulting in a reduction of the achievable endurance cycles. Therefore, applying an optimal voltage serves as an effective strategy to suppress stress and enhance the endurance cycling.

Supplementary Note Figure S5-4 presents the phase transitions occurring due to voltage adjustments, particularly in relation to endurance cycles. In **Supplementary Note Figure S5-4a**, a reference line is drawn at $2P_r$ = 50 μC/cm^2. The experimental data points where $2P_r$ intersects this reference line are labeled a-g. Marks a-c correspond to applied voltages of 16 V, 17 V, and 18 V, respectively, indicating the progression of partial wake-up. Marks d-g represent the degradation phase associated with fatigue, where material performance declines.

The markers in **Supplementary Note Figure S5-4b** correspond directly to those in **Supplementary Note Figure S5-4a**, representing the same conventional $2P_r$ and applied voltage conditions at different endurance cycles. This consistent labeling highlights the correlation between the two figures. The voltage initially increases gradually to bring the conventional $2P_r$ close to the preset $2P_r$. Subsequently, as seen in marks a-c in **Supplementary Note Figure S5-4b**, the applied voltage decreases due to partial wake-up, leading to a reduction in operational voltage. This observation aligns with **Supplementary Note Figure S5-4a**, where the corresponding region confirms the occurrence of partial wake-up. In contrast, marks d-g correspond to the fatigue phase, where degradation becomes more pronounced. This is evident from the decrease in conventional $2P_r$ in **Supplementary Note Figure S5-4a**, while in

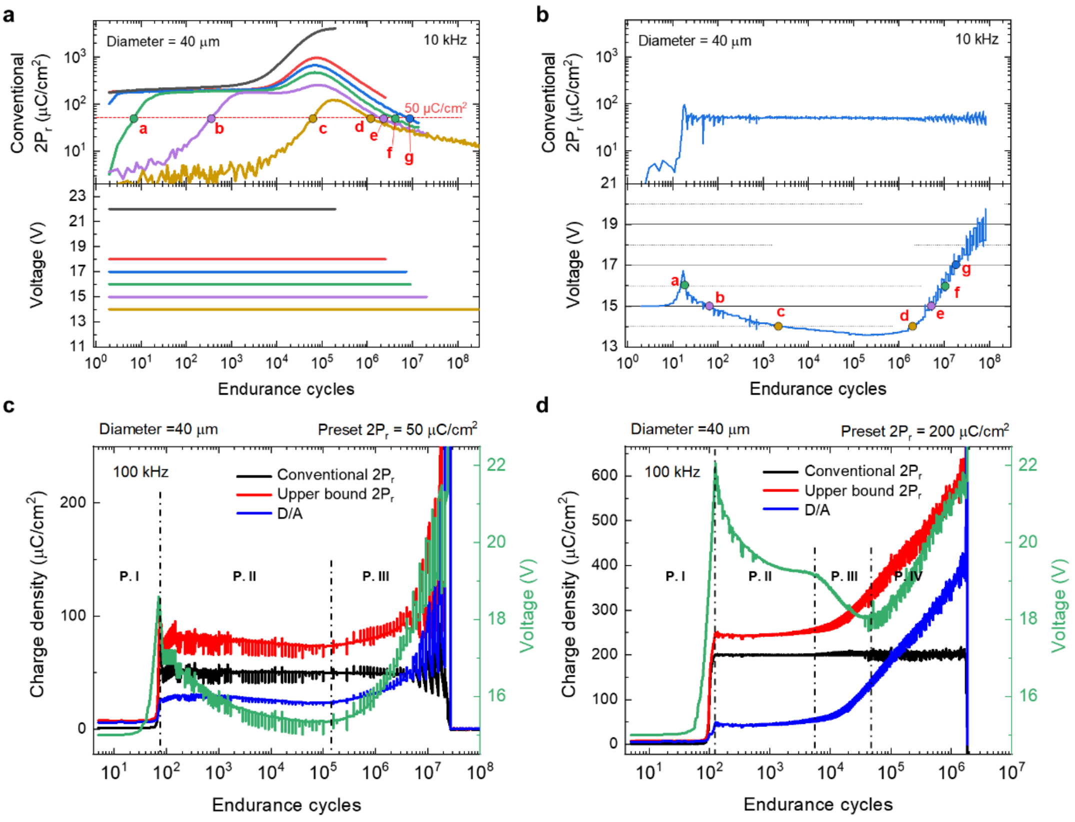

Supplementary Note Figure S5-4 | Endurance test results related to phase transition for a preset $2P_r$ of 50 μC/cm^2. (a) exhibits constant applied voltage pulses with a reference line and **(b)** adjusted applied voltage pulses to maintain a conventional $2P_r$ of 50 μC/cm^2. **(c)** and **(d)** show endurance tests under different preset $2P_r$ values.

Supplementary Note Figure S5-4b, the adjusted applied voltage increases as degradation progresses as the process maintains conventional $2P_r$ close to the preset $2P_r$. These trends indicate that a decreasing voltage corresponds to partial wake-up, whereas an increasing voltage signifies material degradation.

A notable observation is the extended interval between marks c and d. By adjusting the voltage, the preset $2P_r$ level is attained more quickly. As a result, marks a-c in **Supplementary Note Figure S5-4b** appear earlier in the endurance cycle compared to their counterparts in **Supplementary Note Figure S5-4a**, while marks d-g appear later. Notably, the endurance cycle values for d-g in **Supplementary Note Figure S5-4b** are nearly twice those in **Supplementary Note Figure S5-4a**, demonstrating an extended device lifespan with optimized voltage adjustment.

Supplementary Note Figure S5-4c presents the results obtained at 100 kHz, providing a detailed analysis of voltage-dependent phase transitions. Not only conventional $2P_r$ but also the variations of N/A and D/A are plotted together. During P. I of **Supplementary Note Figure S5-4c**, the algorithm

rapidly adjusts the applied voltage to achieve the appropriate initial conditions. Subsequently, in P. II, a continuous decrease in applied voltage is observed, which is attributed to partial wake-up effects. During this phase, both N/A and D/A show a slight decreasing trend, as leakage is suppressed due to

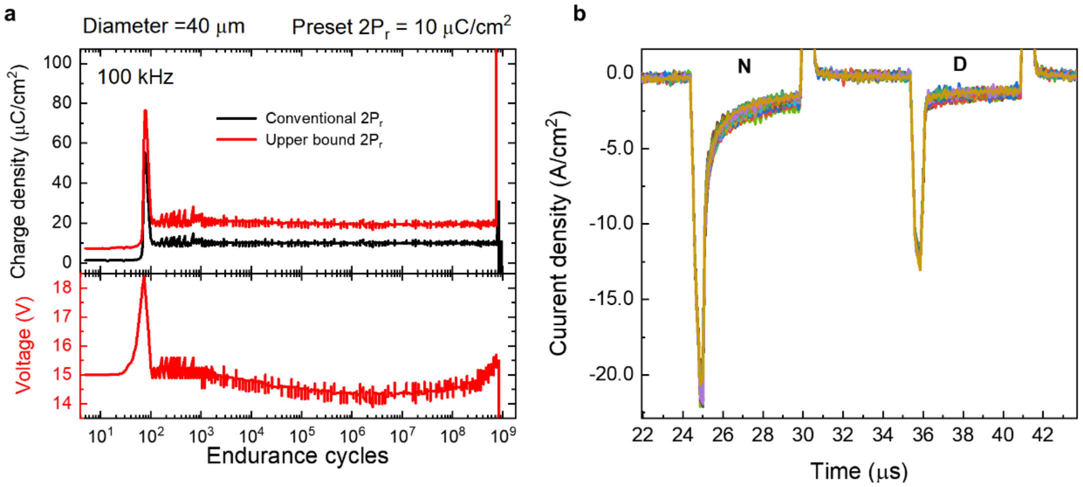

Supplementary Note Figure S5-5 | Endurance test results and N&D current responses with 40 m diameter under 100 kHz to adjust applied voltage to maintain the conventional 2P$_r$ of 10 µC/cm^2.

the reduction in applied voltage. This process is important in delaying ferroelectric breakdown by mitigating electrical stress from the reduced adjusted applied voltage and minimizing current stress resulting from decreased leakage. In P. III, as degradation progresses, leakage increases, leading to a simultaneous rise in both N/A and D/A. Consequently, the applied voltage is also adjusted upward to maintain stable conventional 2P$_r$ switching.

Supplementary Note Figure S5-4d illustrates the voltage adjustment required to maintain full switching (~200 µC/cm^2). In this case, unlike **Supplementary Note Figure S5-4c**, four distinct phases can be identified. In P. I of **Supplementary Note Figure S5-4d**, the process of finding the appropriate voltage proceeds similarly to **Supplementary Note Figure S5-4c**. Following this, in P. II, the applied voltage continues to decrease. During this phase, not only does partial wake-up occur, but the stable phase also continues simultaneously. In P. III, a sharp decrease in applied voltage occurs as N/A and D/A increase, like P. II in **Supplementary Note Figure S5-3a**, indicating a phase where leakage rises rapidly. This results in a voltage drop as the system attempts to maintain a constant conventional 2P$_r$. In P. IV, the applied voltage starts to rise, indicating the onset of the fatigue phase caused by ferroelectric degradation.

Supplementary Note Figures S5-1 to **S5-4** also provide the relation between leakage and conventional 2P$_r$. The leakage charge during the D pulse increases steadily at high cycle counts and

follows the drop in conventional $2P_r$ when the drive voltage is fixed. **Supplementary Note Figures** from **S5-1** to **S5-3** show the final stage of the endurance tests under each fixed voltage condition, corresponding to Phase III (**Supplementary Note Figures S5-1** and **S5-3**) and Phase IV (**Supplementary Note Figure S5-2**). Under constant applied voltage, the growth of leakage indeed lowers the measured conventional $2P_r$. This behavior is consistent with prior reports on ferroelectrics[58-66] where defects and traps create leakage paths along the nitrogen vacancies diverting switching energy and pinning domains[23] and leading to interfacial non ferroelectric layer thickening[67]. However, during the early onset of leakage, such as Phase II (**Supplementary Note Figures S5-1** and **S5-3**) and Phase III (**Supplementary Note Figure S5-2**), Maxwell–Wagner induced Curie–von Schweidler relaxation current[68, 69] can overlap with switching peaks, briefly inflating $2P_r$. Thus, leakage may either overestimate conventional $2P_r$ or drive true polarization loss.

Leakage can either lead to an overestimation or underestimation of the conventional $2P_r$, raising inevitable questions about the accuracy of PUND at high leakage levels. Nevertheless, we consistently observed reproducible current peaks during endurance cycling, which provide direct evidence of ferroelectric dipole switching. Even near the end-of-test, these switching signatures remain clearly detectable, confirming that the devices sustain ferroelectric switching over extended cycling. Therefore, while the real value of $2P_r$ becomes less reliable due to leakage, the persistence of switching peaks validates the endurance results reported here.

Supplementary Note Figure S5-5a shows a diameter of 40 μm with a frequency of 100 kHz and a preset $2P_r$ of 10 $\mu C/cm^2$. In this case, there is little change in the conventional $2P_r$ and upper bound $2P_r$ response throughout the entire endurance test. This indicates that severe leakage does not occur. As a result, additional current stress is minimized. Since only the minimum necessary voltage is applied through the voltage adjustment process, voltage stress is also minimized. Therefore, these minimized stress leads to an increase in endurance. **Supplementary Note Figure S5-5b** shows the N and D current density responses from 136 cycles to 5.85E+08 cycles. It confirms that there is no significant change in this response during the entire endurance test. This means that a very stable response is maintained throughout the entire test.

References

1. J. X. Zheng, M. M. A. Fiagbenu, G. Esteves, P. Musavigharavi, A. Gunda, D. Jariwala, E. A. Stach, R. H. Olsson, Ferroelectric behavior of sputter deposited $Al_{0.72}Sc_{0.28}N$ approaching 5 nm thickness. *Appl. Phys. Lett.,* 122 (22), 222901 (2023)
2. J. Su, S. Fichtner, M. Z. Ghori, N. Wolff, M. R. Islam, A. Lotnyk, D. Kaden, F. Niekiel, L. Kienle, B. Wagner, F. Lofink, Growth of Highly c-Axis Oriented AlScN Films on Commercial Substrates, *Micromachines (Basel)*., 13(5), 783 (2022)
3. V. V. Felmetsger, M. K. Mikhov, Deposition of smooth and highly (111) textured Al bottom electrodes for AlN-based electroacoustic devices, *2012 IEEE International Frequency Control Symposium Proceedings*, Baltimore, MD, USA, pp. 1-4 (2012)
4. S. Wang, V. Dhyani, S. S. Mohanraj, X. Shi, B. Varghese, W. W. Chung, D. Huang, Z. Shiuh Lim, Q. Zeng, H. Liu, X. Luo, V. Leong, N. Li, D. Zhu, CMOS-compatible photonic integrated circuits on thin-film ScAlN. *APL Photonics*, 9 (6), 066109 (2024)
5. R. Nie, S. Shao, Z. Luo, X. Kang, T. Wu, Characterization of Ferroelectric $Al_{0.7}Sc_{0.3}N$ Thin Film on Pt and Mo Electrodes, *Micromachines* 13, no. 10, 1629 (2022)
6. Y. Lu, M. Reusch, N. Kurz, A. Ding, T. Christoph, M. Prescher, L. Kirste, O. Ambacher, A. Žukauskaitė, Elastic modulus and coefficient of thermal expansion of piezoelectric $Al_{1-x}Sc_xN$ (up to x = 0.41) thin films. *APL Mater.*, 6 (7), 076105 (2018)
7. D. Wang, P. Musavigharavi, J. Zheng, G. Esteves, X. Liu, M. M. A. Fiagbenu, E. A. Stach, D. Jariwala, R. H. Olsson III, Sub-Microsecond Polarization Switching in (Al,Sc)N Ferroelectric Capacitors Grown on Complementary Metal–Oxide–Semiconductor-Compatible Aluminum Electrodes. *Phys. Status Solidi RRL*, 15: 2000575 (2021)
8. S. Barth, T. Schreiber, S. Cornelius, O. Zywitzki, T. Modes, H. Bartzsch, High Rate Deposition of Piezoelectric AlScN Films by Reactive Magnetron Sputtering from AlSc Alloy Targets on Large Area, *Micromachines (Basel)*.,13(10), 1561 (2022).
9. D. Drury, K. Yazawa, A. Zakutayev, B. Hanrahan, G. Brennecka, High-Temperature Ferroelectric Behavior of $Al_{0.7}Sc_{0.3}N$, *Micromachines (Basel).* May 31;13(6), 887 (2022)
10. X. Liu, J. Zheng, D. Wang, P. Musavigharavi, E. A. S., R. Olsson, D. Jariwala, Aluminum scandium nitride-based metal–ferroelectric–metal diode memory devices with high on/off ratios. *Appl. Phys. Lett.*, 118 (20), 202901 (2021)
11. P. Wang, D. Wang, S. Mondal, Z. Mi, Ferroelectric N-polar ScAlN/GaN heterostructures grown by molecular beam epitaxy. *Appl. Phys. Lett.*, 121 (2), 023501 (2022)
12. KH. Kim, Z. Han, Y. Zhang, P. Musavigharavi, J. Zheng, D. K. Pradhan, E. A. Stach, R. H. Olsson III, D. Jariwala, *ACS Nano* 18 (24), 15925-15934 (2024)

13. Z. Hu, H. Cho, R. K. Rai, K. Bao, Y. Zhang, Z. Qu, Y. He, Y. Ji, C. Leblanc, KH Kim, Z. Han, Z. Qiu, X. Du, E. A. Stach, R. Olsson, D. Jariwala, Demonstration of Highly Scaled AlScN Ferroelectric Diode Memory with a Storage Density of >100 Mbit/mm^2, *Nano Letters 25* (37), 13748-13755 (2025)
14. D. Zhao, T. Lenz, G. H. Gelinck, P. Groen, D. Damjanovic, D. M. de Leeuw, I. Katsouras, Depolarization of multidomain ferroelectric materials. Nat Commun **10**, 2547 (2019)
15. B. Darinskii, A. Sidorkin, A. Sigov, N. Popravko, Influence of Depolarizing Fields and Screening Effects on Phase Transitions in Ferroelectric Composites. Materials (Basel). Jan 6;11(1):85 (2018)
16. S. Fichtner, N. Wolff, F. Lofink, L. Kienle, B. Wagner, AlScN: A III-V semiconductor based ferroelectric, *J. Appl. Phys.*, **125**, 114103 (2019)
17. W. Zhu, J. Hayden, F. He, JI Yang, P. Tipsawat, M. D. Hossain, JP Maria, S. Trolier-McKinstry, Strongly temperature dependent ferroelectric switching in AlN, Al1-xScxN, and Al1-xBxN thin films., Appl. Phys. Lett., 119 (6): 062901., (2021)
18. V. Gund, B. Davaji, H. Lee, J. Casamento, H. G. Xing, D. Jena, A. Lal, Towards Realizing the Low-Coercive Field Operation of Sputtered Ferroelectric ScxAl1-xN, 2021 21st International Conference on Solid-State Sensors, Actuators and Microsystems (Transducers), Orlando, FL, USA, 1064-1067, (2021)
19. D. Wang, P. Wang, B. Wang, Z. Mi, Fully epitaxial ferroelectric ScGaN grown on GaN by molecular beam epitaxy., Appl. Phys. Lett., 119, 111902, (2021)
20. S. Song, D. K. Pradhan, Z. Hu, Y. Zhang, R. N. Keneipp, M. A. Susner, P. Bhattacharya, M. Drndić, R. H. Olsson III, D. Jariwala, Observation of giant remnant polarization in ultrathin AlScN at cryogenic temperatures, arXiv:2503.19491 (2025)
21. L. Chen, C. Liu, H. K. Lee, B. Varghese, R. W. F. Ip, M. Li, Z. J. Quek, Y. Hong, W. Wang, W. Song, H. Lin, Y. Zhu, Demonstration of 10 nm Ferroelectric $Al_{0.7}Sc_{0.3}N$-Based Capacitors for Enabling Selector-Free Memory Array, *Materials*, *17*(3), 627 (2024)
22. K. D. Kim, Y. B. Lee, S. H. Lee, I. S. Lee, S. K. Ryoo, S. Byun, J. H. Lee, H. Kim, H. W. Park, C. S. Hwang, Evolution of the Ferroelectric Properties of AlScN Film by Electrical Cycling with an Inhomogeneous Field Distribution. *Adv. Electron. Mater*, 0, 2201142 (2023)
23. K. D. Kim, Y. B. Lee, S. H. Lee, I. S. Lee, S. K. Ryoo, S. Y. Byun, J. H. Lee, C. S. Hwang, Impact of operation voltage and NH_3 annealing on the fatigue characteristics of ferroelectric AlScN thin films grown by sputtering, *Nanoscale*,15, 16390-16402 (2023)
24. X. Li, P. Srivari, E. Paasioa, S. Majumdar, Understanding fatigue and recovery mechanisms in $Hf_{0.5}Zr_{0.5}O_2$ capacitors for designing high endurance ferroelectric memory and neuromorphic hardware, *Nanoscale*,17, 6058-6071 (2025)

25. H. Bohuslavskyi, K. Grigoras, M. Ribeiro, M. Prunnila, S. Majumdar, Ferroelectric $Hf_{0.5}Zr_{0.5}O_2$ for Analog Memory and In-Memory Computing Applications Down to Deep Cryogenic Temperatures. *Adv. Electron. Mater.*, 10, 2300879 (2024)
26. Z. Gao, Y. Luo, S. Lyu, Y. Cheng, Y. Zheng, Q. Zhong, Identification of Ferroelectricity in a Capacitor With Ultra-Thin (1.5-nm) $Hf_{0.5}Zr_{0.5}O_2$ Film, *IEEE Electron Device Letters*, vol. 42, no. 9, pp. 1303-1306, (2021)
27. J. Bouaziz, P. R. Romeo, N. Baboux, B. Vilquin, Huge Reduction of the Wake-Up Effect in Ferroelectric HZO Thin Films, *ACS Applied Electronic Materials* 1 (9), 1740-1745 (2019)
28. Y. Cao, W. Zhang Y. Li, Hafnium-doped zirconia ferroelectric thin films with excellent endurance at high polarization, *Nanoscale*,15, 1392-1401 (2023)
29. M. Akiyama, T. Kamohara, K. Kano, A. Teshigahara, Y. Takeuchi, N. Kawahara, Enhancement of Piezoelectric Response in Scandium Aluminum Nitride Alloy Thin Films Prepared by Dual Reactive Cosputtering, *Adv. Mater.*, **21**, 593–596 (2009)
30. M. Lanza, H.-S. P. Wong, E. Pop, D. Ielmini, D. Strukov, B. C. Regan, L. Larcher, M. A. Villena, J. J. Yang, L. Goux, A. Belmonte, Y. Yang, F. M. Puglisi, J. Kang, B. Magyari-Köpe, E. Yalon, A. Kenyon, M. Buckwell, A. Mehonic, A. Shluger, H. Li, T.-H. Hou, B. Hudec, D. Akinwande, R. Ge, S. Ambrogio, J. B. Roldan, E. Miranda, J. Suñe, K. L. Pey, X. Wu, N. Raghavan, E. Wu, W. D. Lu, G. Navarro, W. Zhang, H. Wu, R. Li, A. Holleitner, U. Wurstbauer, M. C. Lemme, M. Liu, S. Long, Q. Liu, H. Lv, A. Padovani, P. Pavan, I. Valov, X. Jing, T. Han, K. Zhu, S. Chen, F. Hui, Y. Shi, Recommended Methods to Study Resistive Switching Devices, *Adv. Electron. Mater.*, 5, 1800143 (2019)
31. Y. Zuo, H. Lin, J. Guo, Y. Yuan, H. He, Y. Li, Y. Xiao, X. Li, K. Zhu, T. Wang, X. Jing, C. Wen, M. Lanza, Effect of the Pressure Exerted by Probe Station Tips in the Electrical Characteristics of Memristors. *Adv. Electron. Mater.*, 6, 1901226 (2020)
32. K. N. Tu, Yingxia Liu, Menglu Li, Effect of Joule heating and current crowding on electromigration in mobile technology. *Appl. Phys. Rev.,* 4 (1): 011101 (2017)
33. E. Tokumitsu, N. Tanisake, H. Ishiwara, Partial Switching Kinetics of Ferroelectric $PbZr_xTi_{1-x}O_3$ Thin Films Prepared by Sol-Gel Technique, Jpn. J. Appl. Phys., 33, 5201, (1994)
34. C. Alessandri, P. Pandey, A. Abusleme, A. Seabaugh, Switching Dynamics of Ferroelectric Zr Doped HfO_2, IEEE Electron Device Letters, 39, 1780-1783, (2018)
35. S. Oh, H. Hwang, I. K. Yoo, Ferroelectric materials for neuromorphic computing, APL Mater., 7, 091109., (2019)
36. R. Guido, H. Lu, P. D. Lomenzo, T. Mikolajick, A. Gruverman, U. Schroeder, Kinetics of Nto M-Polar Switching in Ferroelectric Al1−xScxN Capacitors., Adv. Sci., 11, 2308797., (2024)

37. K. Yazawa, J. Hayden, JP Maria, W. Zhu, S. Trolier-McKinstry, A. Zakutayev, G. L. Brennecka, Mater. Horiz., 10, 2936-2944, (2023)
38. Z. Tang, G. Esteves, R. H. Olsson III, Sub-quarter micrometer periodically poled $Al_{0.68}Sc_{0.32}N$ for ultra-wideband photonics and acoustic devices, *J. Appl. Phys.* 134, 114101 (2023)
39. H. Lu, G. Schönweger, A. Petraru, H. Kohlstedt, S. Fichtner, A. Gruverman, Domain Dynamics and Resistive Switching in Ferroelectric $Al_{1-x}Sc_xN$ Thin Film Capacitors, *Adv. Funct. Mater.*, 34, 2315169 (2024)
40. H. Lu, G. Schönweger, N. Wolff, Z. Ding, A. Petraru, I. Streicher, H. Kohlstedt, C. Kübel, S. Leone, L. Kienle, S. Fichtner, A. Gruverman, $Al_{1-x}Sc_xN$-Based Ferroelectric Domain-Wall Memristors. *Adv. Funct. Mater.*, 2503143 (2025)
41. C.W. Lee, K. Yazawa, A. Zakutayev, G.L. Brennecka, P. Gorai, Switching it up: new mechanisms revealed in wurtzite-type ferroelectrics, *Sci. Adv.*, 10 (20) (2024)
42. S. Fichtner, G. Schönweger, CW. Lee, K. Yazawa, P. Gorai, G. L. Brennecka, Polarization and domains in wurtzite ferroelectrics: Fundamentals and applications. *Appl. Phys. Rev.*, 12 (2): 021310 (2025)
43. S. Calderon V, J. Hayden, S. M. Baksa, W. Tzou, S. Trolier-McKinstry, I. Dabo, JP. Maria, Elizabeth C. Dickey, Atomic-scale polarization switching in wurtzite ferroelectrics, *Science,* **380**,1034-1038 (2023)
44. X. J. Lou, Polarization fatigue in ferroelectric thin films and related materials, *J. Appl. Phys.* **105**, 024101 (2009)
45. A. K. Tagantsev, I. Stolichnov, N. Setter, J. S. Cross, M. Tsukada, Non-Kolmogorov-Avrami switching kinetics in ferroelectric thin films, *Phys. Rev. B*, **66**, 214109 (2002)
46. Y. Kim, HH. Han, W. Lee, S. Baik, D. Hesse, M. Alexe, Non-Kolmogorov−Avrami−Ishibashi Switching Dynamics in Nanoscale Ferroelectric Capacitors *Nano Letters* 10 (4), 1266-1270 (2010)
47. Y. Ahn, J. Y. Son, Activation field-driven domain wall dynamics of nanobits in ferroelectric $Al_{0.7}Sc_{0.3}N$ thin films, *Journal of Alloys and Compounds*, 1035, 5, 181529 (2025)
48. R. Bulanadi, K. Cordero-Edwards, P. Tückmantel, S. Saremi, G. Morpurgo, Q. Zhang, L. W. Martin, V. Nagarajan, P. Paruch, Interplay between Point and Extended Defects and Their Effects on Jerky Domain-Wall Motion in Ferroelectric Thin Films, *Phys. Rev. Lett.* **133**, 106801 (2024)
49. CW. Lee, N. U. Din, G. L. Brennecka, P. Gorai, Defects and oxygen impurities in ferroelectric wurtzite $Al_{1-x}Sc_xN$ alloys. *Appl. Phys. Lett.*, 125 (2): 022901 (2024)
50. SH. Teng, A. Dimou, B. Udofia, M. Ghasemi, M. Stricker, A. Grünebohm, Control of ferroelectric domain wall dynamics by point defects: Insights from *ab initio* based

simulations. *J. Appl. Phys.* 137 (15): 154103 (2025)

51. G. Catalan, J. Seidel, R. Ramesh, J. F. Scott, Domain wall nanoelectronics, *Rev. Mod. Phys.* **84**, 119 (2012)
52. Y. Zhan, Q. Zhu, B. Tian, C. Duan, New-Generation Ferroelectric AlScN Materials, *Nanomicro Lett.*, 16:227 (2024)
53. M. Y. Gureev, P. Mokrý, A. K. Tagantsev, N. Setter, Ferroelectric charged domain walls in an applied electric field, *Phys. Rev. B* **86**, 104104 (2012)
54. M. T. Do, N. Gauquelin, M. D. Nguyen, F. Blom, J. Verbeeck, G. Koster, E. P. Houwman, G. Rijnders, Interface degradation and field screening mechanism behind bipolar-cycling fatigue in ferroelectric capacitors. *APL Mater.*, 9 (2): 021113 (2021)
55. D. R. Småbråten, T. S. Holstad, D. M. Evans, Z. Yan, E. Bourret, D. Meier, S. M. Selbach, Domain wall mobility and roughening in doped ferroelectric hexagonal manganites, *Phys. Rev. Research* **2**, 033159 (2020)
56. P. S. Bednyakov, T. Sluka, A. K. Tagantsev, D. Damjanovic, N. Setter, Formation of charged ferroelectric domain walls with controlled periodicity, *Scientific Reports*, volume 5, Article number: 15819 (2015)
57. T. Hwang, W. Aigner, T. Metzger, A. C. Kummel, K. Cho, First-Principles Understanding on the Formation of Inversion Domain Boundaries of Wurtzite AlN, AlScN, and GaN, *ACS Applied Electronic Materials* 6 (5), 3257-3263(2024)
58. X. Cheng, C. Zhou, B. Lin, Z Yang, S Chen, K. H.L. Zhang, Z. Chen, Leakage mechanism in ferroelectric $Hf_{0.5}Zr_{0.5}O_2$ epitaxial thin films, *Applied Materials Today*, 32, 101804 (2023)
59. S. S. Fields, S. W. Smith, S. T. Jaszewski, T. Mimura, D. A. Dickie, G. Esteves, M. D. Henry, S. L. Wolfley, P. S. Davids, J. F. Ihlefeld, Wake-up and fatigue mechanisms in ferroelectric $Hf_{0.5}Zr_{0.5}O_2$ films with symmetric RuO_2 electrodes. *J. Appl. Phys.,* **130** (13), 134101 (2021)
60. E. D. Grimley, T. Schenk, X. Sang, M. Pešić, U. Schroeder, T. Mikolajick, J. M. LeBeau, Structural Changes Underlying Field-Cycling Phenomena in Ferroelectric HfO_2 Thin Films. *Adv. Electron. Mater.*, 2, 1600173 (2016)
61. Y. Cheng, Z. Gao, K. H. Ye, H. W. Park, Y. Zheng, Y. Zheng, J. Gao, M. H. Park, JH Choi, KH Xue, C. S. Hwang, H. Lyu, Reversible transition between the polar and antipolar phases and its implications for wake-up and fatigue in HfO_2-based ferroelectric thin film. *Nat Commun* **13**, 645 (2022).
62. S. Li, D. Zhou, Z. Shi, M. Hoffmann, T. Mikolajick, U. Schroeder, Involvement of Unsaturated Switching in the Endurance Cycling of Si-doped HfO_2 Ferroelectric Thin Films. *Adv. Electron. Mater.*, 6, 2000264 (2020)

63. C. Wang, H. Qiao, Y. Kim, Perspective on the switching behavior of HfO_2-based ferroelectrics. *J. Appl. Phys.*, 129 (1), 010902 (2021)
64. X. Li, P. Srivari, E. Paasio, S. Majumda, Understanding fatigue and recovery mechanisms in $Hf_{0.5}Zr_{0.5}O_2$ capacitors for designing high endurance ferroelectric memory and neuromorphic hardware, *Nanoscale*, 17, 6058-6071 (2025)
65. A. Mallick, M. K. Lenox, T. E. Beechem, J. F. Ihlefeld, N. Shukla, Oxygen vacancy contributions to the electrical stress response and endurance of ferroelectric hafnium zirconium oxide thin films. *Appl. Phys. Lett.*, 122 (13), 132902 (2023)
66. K. Yazawa, C. Evans, E. C. Dickey, M. B. Tellekamp, G. L. Brennecka, A. Zakutayev, Low leakage current in heteroepitaxial $Al_{0.7}Sc_{0.3}N$ ferroelectric films on GaN, *Phys. Rev. Applied* 23, 014036 (2025)
67. SL. Tsai, T. Hoshii, H. Wakabayashi, K. Tsutsui, TK. Chung, E. Y. Chang, K. Kakushima, Field cycling behavior and breakdown mechanism of ferroelectric $Al_{0.78}Sc_{0.22}N$ films, *Jpn. J. Appl. Phys.* **61** SJ1005 (2022)
68. J Liu, CG. Duan, WG. Yin, W. N. Mei1, R. W. Smith, J. R. Hardy, Large dielectric constant and Maxwell-Wagner relaxation in $Bi_{2/3}Cu_3Ti_4O_{12}$, *Phys. Rev. B* **70**, 144106 (2004)
69. R. Bouregba, On the origin of polarization fatigue and Curie–von Schweidler relaxation current in $Pb(Zr_x,Ti_{1-x})O_3$ ferroelectric thin films: A unique mechanism based on charge trapping by interface defects, *J. Appl. Phys.* 133, 014101 (2023)
